# Whole Genome Sequencing Provides an Added Value to the Investigation of Staphylococcal Food Poisoning Outbreaks

**DOI:** 10.3389/fmicb.2021.750278

**Published:** 2021-11-02

**Authors:** Stéphanie Nouws, Bert Bogaerts, Bavo Verhaegen, Sarah Denayer, Lasse Laeremans, Kathleen Marchal, Nancy H. C. Roosens, Kevin Vanneste, Sigrid C. J. De Keersmaecker

**Affiliations:** ^1^Transversal Activities in Applied Genomics, Sciensano, Brussels, Belgium; ^2^IDLab, Department of Information Technology, Ghent University - IMEC, Ghent, Belgium; ^3^Department of Plant Biotechnology and Bioinformatics, Ghent University, Ghent, Belgium; ^4^National Reference Laboratory for Foodborne Outbreaks (NRL-FBO) and for Coagulase Positive Staphylococci (NRL-CPS), Foodborne Pathogens, Sciensano, Brussels, Belgium; ^5^Organic Contaminants and Additives, Sciensano, Brussels, Belgium; ^6^Department of Genetics, University of Pretoria, Pretoria, South Africa

**Keywords:** whole genome sequencing, *Staphylococcus aureus*, staphylococcal food poisoning (SFP), outbreak investigation, enterotoxin gene profiling, relatedness determination, DNA extraction kit

## Abstract

Through staphylococcal enterotoxin (SE) production, *Staphylococcus aureus* is a common cause of food poisoning. Detection of staphylococcal food poisoning (SFP) is mostly performed using immunoassays, which, however, only detect five of 27 SEs described to date. Polymerase chain reactions are, therefore, frequently used in complement to identify a bigger arsenal of SE at the gene level (*se*) but are labor-intensive. Complete *se* profiling of isolates from different sources, i.e., food and human cases, is, however, important to provide an indication of their potential link within foodborne outbreak investigation. In addition to complete *se* gene profiling, relatedness between isolates is determined with more certainty using pulsed-field gel electrophoresis, *Staphylococcus* protein A gene typing and other methods, but these are shown to lack resolution. We evaluated how whole genome sequencing (WGS) can offer a solution to these shortcomings. By WGS analysis of a selection of *S. aureus* isolates, including some belonging to a confirmed foodborne outbreak, its added value as the ultimate multiplexing method was demonstrated. In contrast to PCR-based *se* gene detection for which primers are sometimes shown to be non-specific, WGS enabled complete *se* gene profiling with high performance, provided that a database containing reference sequences for all *se* genes was constructed and employed. The custom compiled database and applied parameters were made publicly available in an online user-friendly interface. As an all-in-one approach with high resolution, WGS additionally allowed inferring correct isolate relationships. The different DNA extraction kits that were tested affected neither *se* gene profiling nor relatedness determination, which is interesting for data sharing during SFP outbreak investigation. Although confirming the production of enterotoxins remains important for SFP investigation, we delivered a proof-of-concept that WGS is a valid alternative and/or complementary tool for outbreak investigation.

## Introduction

Through the production of staphylococcal enterotoxins (SEs), *Staphylococcus aureus* is globally one of the most common causative agents responsible for food poisoning outbreaks [European Food Safety Authority (EFSA) and European Centre for Disease Prevention and Control (ECDC), 2021]. Detection of staphylococcal food poisoning (SFP) is, therefore, mainly based on the detection of SEs in food leftovers, the enumeration of >10^5^ *S. aureus* CFU/g food, and the isolation of *S. aureus* from the poisoned food source and affected human cases (Hennekinne et al., 2010).

For SE detection, commercial kits using immunological methods have been developed, such as the automated VIDAS^®^ immunoanalyzer or the Staphylococcal Enterotoxin Reversed Passive Latex Agglutination (SET-RPLA) kit, and are widely used by National Reference Laboratories (NRLs) and Centers, often under standardized ISO 19020:2017 environments (Hennekinne et al., 2010). However, these kits only allow detection of at most five SEs (the classical five SE: SEA to SEE), whereas an arsenal of 27 different enterotoxins is described to date (Merda et al., 2020). Moreover, due to insertion, deletion, duplication, and recombination events in their encoding genes, new SEs [such as SELV and SELU arising from fusion between *sem* and *sei* (Thomas et al., 2006) and Ψ*ent1* and Ψ*ent2* pseudogenes (Letertre et al., 2003b; Thomas et al., 2006), respectively] and SE variants (such as SEC_1__–__4_, SEC_bovine_, SEC_ovine_, SELU2, and others) are continuously generated. Many of the described SEs are determined to exhibit emetic properties [in alphabetical order: SEA to SEE, SEG to SEI, SEK to SET, and SEY (Spaulding et al., 2013; Johler et al., 2015; Wilson et al., 2018; Zhang et al., 2018; Aung et al., 2020)]. Moreover, it is most likely that multiple of these toxins are produced during SFP and play a (contributing) role in the provoked illness (Umeda et al., 2017; Fisher et al., 2018). Therefore, it is important to detect all toxins involved, not only during SFP outbreak investigation, but also during SFP surveillance, to assess the possible risk of strains isolated from food matrices in view of illness prevention. Because the development of new immunological assays remains difficult (Fisher et al., 2018), molecular methods, such as polymerase chain reactions (PCRs) are frequently used to detect SE encoding genes (*se*) (Fisher et al., 2018). Although no information on the eventual production of the toxin is obtained, detection at the genotypic level demonstrates the potential for toxin production. In response, many PCR primer pairs have been developed detecting the different *se* genes (for references, see Supplementary Table 1). However, the fact that multiple primer sets per se gene are described across the literature and not all are exhaustively investigated for their specificity impedes the selection of the most appropriate primers for routine applications. Indeed, the high sequence similarity between *se* (pseudo)genes and mutations in the primer binding sites of *se* variants increases the risk of false positives and negatives, respectively. Moreover, despite efforts in developing multiplex PCR assays targeting multiple *se* genes per assay (Becker et al., 1998; Schmitz et al., 1998; Monday and Bohach, 1999; Mehrotra et al., 2000; Sharma et al., 2000; Letertre et al., 2003a; Shylaja et al., 2010; Nagaraj et al., 2014), detecting the full arsenal of *se* genes currently described (from now on referred to as “complete *se* gene profiling”) remains a labor-intensive and time-consuming process.

Complete *se* gene profiling can, however, also provide an initial indication on the potential link between the contaminated food source and the human case(s) (Denayer et al., 2017). Yet, because food sources are sometimes contaminated with several *S. aureus* strains, the level of isolate discrimination based on the *se* gene profile might not be sufficient. Indeed, accurate epidemiological typing methods are a prerequisite to distinguish between isolated strains and aiding in identifying the causal strain for SFP outbreaks. For this purpose, pulsed-field gel electrophoresis (PFGE), multiple-locus variable number of tandem repeats analysis (MLVA), and phage typing are still regarded as the gold standard to study isolate relatedness (Blair and Williams, 1961; Wildemauwe et al., 2004; Roussel et al., 2015). However, these methods have shown some issues with interpretation and/or interlaboratory comparison of obtained profiles (Cookson et al., 1996; Murchan et al., 2003; Price et al., 2013). Therefore, DNA sequence-based methods, such as *Staphylococcus* protein A gene (*spa*) typing and multilocus sequence typing (MLST) are frequently applied as well because their analysis is less ambiguous. Also for MLVA, DNA sequencing of alleles has been used to increase the initially limited transferability of obtained data by confirming the deduced number of repeats (Schouls et al., 2009). However, all these conventional methods are recently shown to struggle in distinguishing closely related *S. aureus* isolates (Moore et al., 2015; Roussel et al., 2015; Cunningham et al., 2017; Cremers et al., 2020).

Because whole genome sequencing (WGS) analyzes the entire genome of bacteria, it enables complete genotypic characterization of isolates. Thanks to its unparalleled resolution standing out compared with conventional methods (Anderson et al., 2014; Moore et al., 2015; Cunningham et al., 2017; Cremers et al., 2020), it has moreover become the ultimate tool for inferring phylogenetic relationships between bacterial isolates, using core genome MLST (cgMLST) or single nucleotide polymorphism (SNP) analysis. Although WGS was already used to analyze Methicillin-resistant *S. aureus* strains responsible for nosocomial infections (Anderson et al., 2014; Moore et al., 2015; Peacock and Paterson, 2015; Cunningham et al., 2017; Sabat et al., 2017), it has been scarcely applied for SFP investigation despite the advantages highlighted above (Merda et al., 2020; Schwendimann et al., 2021).

For WGS to be applied in the analysis of *S. aureus* isolates for se gene profiling, it is important to have a DNA sample representative for the isolate so that WGS data are comparable between (inter)national laboratories. Data sharing is indeed indispensable in the scope of outbreak investigations. For *S. aureus*, this means that the extracted genome must contain DNA of mobile genetic elements, such as plasmids, as they often harbor *se* genes (Argudín et al., 2010). Some frequently used, commercial DNA extraction kits have, however, been described as having decreased plasmid extraction performances (Becker et al., 2016; Nouws et al., 2020a), which can potentially impact complete *se* gene profiling. The successful integration of WGS-based *se* gene profiling is, additionally, hindered by the lack of comprehensive and/or freely and easily accessible databases. Indeed, commonly used databases for virulence gene detection in *S. aureus* do not contain or correctly annotate the full arsenal of *se* genes currently described, are not publicly available through an easily accessible online resource, and/or are not always linked to a user-friendly interface to query WGS data for *se* genes (Joensen et al., 2015; Liu et al., 2019; Sayers et al., 2019; Merda et al., 2020).

This study aimed to assess whether WGS would have an added value for application in the investigation of SFP outbreaks. For this purpose, WGS was used for *se* gene detection with a custom database [based on the frequently used Virulence Factor Database (VFDB)] of in-house sequenced isolates, complemented with publicly available WGS data. The determined *se* profiles were compared with those previously obtained with conventional methods supplemented with *in silico* PCR. Different DNA extraction kits were moreover compared for their appropriateness to use for WGS-based *se* gene profiling. The performance of WGS in *S. aureus* isolate relatedness determination and the potential influence of using different DNA extraction kits on this analysis was evaluated by comparison with *a priori* known relationships.

## Materials and Methods

### Selected Isolates and Their Characteristics

#### Criteria for Isolate Selection

To evaluate the performance of WGS in determining *se* gene profiles, isolates (Table 1) were selected to cover a repertory as extensive as possible of different *se* genes. For each of the enterotoxins (genes) whose presence was assessed previously, an in-house available isolate was included, i.e., *sea* to *see*, *seg* to *selj*, *sep*, and *ser* as determined by the multiplex PCR of the European Union Reference Laboratory for Coagulase Positive Staphylococci (EURL-CPS) (Roussel et al., 2015) at genotypic level and/or SEA to SEE as determined by the immunoassays SET-RPLA (detection and separate typing of SEA to SED) and VIDAS (detection of SEA to SEE) analyses at the phenotypic level. Isolates that were part of an SFP outbreak [(Denayer et al., 2017), i.e., outbreak A], previously confirmed based on PFGE profiles, were also included to allow investigating if WGS has an added value in resolving the SFP outbreak as proof of concept. All isolates were provided by the Belgian NRL for Foodborne Outbreaks (NRL-FBO) and Coagulase Positive Staphylococci (NRL-CPS), from which two isolates were initially received from the EURL-CPS as reference strains, i.e., TIAC3971 (S-6) and TIAC3972 (FRI-362).

**TABLE 1:** Characteristics of the selected in-house isolates and isolates for which WGS data were publicly available.

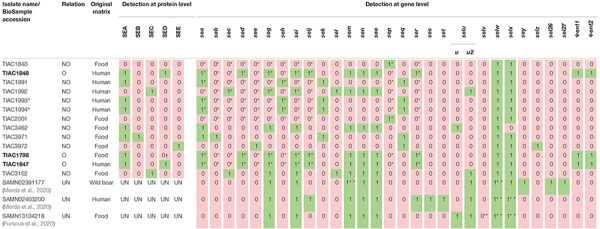

The table contains an overview of all characteristics of the selected isolates for this study (in-house isolates have a name starting with “TIAC,” and publicly available data are listed with their NCBI BioSample accession), the relatedness between the isolates as determined previously with PFGE {indicated as being part of an outbreak (O; and isolate name in bold) [(Denayer et al., 2017); Outbreak A], not being part of an outbreak (NO), or unknown (UN)}, their origin (“original matrix”), and results on their determined SE/*se* profile. TIAC1993 and TIAC1994 were isolated from nose and throat samples, respectively, from the same person (indicated with “°”). TIAC3971 and TIAC3972 are reference strains S-6 (Lopes et al., 1993) and FRI-326 (Bergdoll et al., 1971), respectively, sent by the EURL-CPS. Results of the immunological assays performed in routine are shown if known (UN: unknown), i.e., “Detection at protein level.” Results on the previously performed multiplex PCR (targeted genes are indicated with “*”), *in silico* PCR (this study), and previously in literature reported WGS analysis [discrepant results with the *in silico* PCR are indicated with “**” (Fursova et al., 2020; Merda et al., 2020)] are indicated as “Detection at gene level.” Toxin or gene presence is indicated with “1” in a green box, absence with “0” in a pink box. Subtyping of *se* variants was not performed with the routine analyses. Only when *selu* or *selu2* were detected with *in silico* PCR, their presence was further investigated through comparing the amplicon sequence with those of *selu* (Genbank reference AY158703.1), *selu2* (Genbank reference MN450302.1), and Ψ*ent1-2* (Genbank reference MN450303.1) NCBI reference sequences. The *in silico* PCR results were fully congruent with those obtained from the previously performed multiplex PCR. The depicted *in silico* PCR result was only discrepant for some genes (**) in comparison with the previously reported WGS-based *se* profile (Fursova et al., 2020; Merda et al., 2020), i.e., “1**” in a green box indicated the gene was present with *in silico* PCR but not reported by the previous study, whereas “0**” in a pink box indicated the gene was identified as absent with *in silico* PCR but was detected by the previous study. † SE not detected with SET-RPLA but detected with the multiplex PCR. The result of the *in silico* PCR confirmed the result of the multiplex PCR and, thus, the presence of *sed* in TIAC1798.

For *se* genes not covered within the selected isolates, public WGS raw reads and/or assemblies of *S. aureus* isolates were incorporated (Table 1) to cover the complete arsenal of *se* genes. Accession numbers of the pubic WGS data employed in this study are provided in Supplementary Table 2.

#### Ethics Approval

This study includes *S. aureus* strains isolated from human feces or swabs from human cases and food matrices. Besides the origin of the strain, no human data were applied in this study, and thus, no ethical approval or written informed consent was required.

#### Determining the Complete *se* Gene Profile

Because no information concerning the presence/absence of *se*/SE other than those tested with the EURL-CPS multiplex PCR and/or SET-RPLA/VIDAS commercial kits was initially available (i.e., limited to a maximum of 11 out of the 27 *se*/SE), *in silico* PCR was performed to obtain the complete *se* gene profile of each isolate. The same was done for the isolates for which publicly available WGS data were used as a confirmation of the reported *se* genes in the corresponding publications [(Fursova et al., 2020; Merda et al., 2020); see Table 1 and Supplementary Table 2]. For this *in silico* PCR, the literature was searched to collect all reported conventional PCR primer sets (n: 155) for detection of 27 *se* genes and two pseudogenes (Supplementary Table 1).

Each primer pair described in the literature was checked for its specificity to the target gene (i.e., no aspecific detection of other staphylococcal genes with similar amplicon size) and, if not yet known, its amplicon size, by aligning the primer pairs against the NCBI nucleotide collection (nt) for *S. aureus* (taxid: 1280) using the Primer-BLAST tool of NCBI (Ye et al., 2012) through its online resource^1^ (see Supplementary Data). Besides the criteria previously described (Vanneste et al., 2018), default parameters were applied that assessed whether a primer pair allowed the formation of an *in silico* amplicon.

Based on the investigated specificity of all primer sets, 89.7% of the complete list of collected primers were used for the *in silico* PCR. For this purpose, these 139 primer pairs were aligned against the assemblies of the 13 in-house sequenced *S. aureus* isolates (see Supplementary Data) processed with the GenElute Bacterial gDNA kit and the downloaded assemblies of the three isolates for which WGS data were publicly available, using Primer-BLAST. A gene was considered present when at least one of the gene-specific primer pairs yielded an *in silico* product with correct amplicon size according to the preset criteria. Only when a gene was not detected by any of the primer sets was it considered absent.

Because conventional PCR does not enable discriminating between *selu*, its variant *selu2*, and the pseudogenes Ψ*ent1* and Ψ*ent2* due to high-sequence similarity (Heymans et al., 2010; Liang et al., 2016), their detection is generally followed by DNA Sanger sequencing for confirmation (Collery and Smyth, 2007). Therefore, when the primer pairs indicated the presence of *selu*, *selu2*, or Ψ*ent1-2*, *in silico* PCR was repeated using a primer pair [forward: 5′– TGA TAA TTA GTT TTA ACA CTA AAA TGC G-3′; reverse: 5′– CGT CTA ATT GCC ACG TTA TAT CAG T-3′; (Letertre et al., 2003b)] targeting the complete gene length. The amplicon sequence was extracted and aligned against those of NCBI sequences (see Supplementary Figure 1) harboring *selu* (Genbank reference AY158703.1), *selu2* (Genbank reference MN450302.1), and Ψ*ent1-2* (Genbank reference MN450303.1) using CLC Sequence Viewer 8.0 to type the specific gene based on similarity with the reference sequences.

### DNA Preparation and Quality Control

All 13 in-house sequenced isolates were preserved in a glycerol-brain heart infusion (BHI) broth stock (40.0%) at –80°C until analysis. A loopful of each stock was grown overnight (16 h at 37°C) on nutrient agar plates, and a single colony was then inoculated in 10 ml of BHI and grown overnight with shaking at 37°C and 200 rpm.

DNA extraction was performed on all 13 cultures and a blank sample (BHI incubated overnight) using the GenElute Bacterial Genomic DNA (gDNA) kit (Sigma-Aldrich, Missouri, United States) according to the manufacturer’s protocol for Gram positive bacteria. Three of the 13 isolates (TIAC1798, TIAC1847, and TIAC3152) selected based on previously determined presence of *se*/SE exclusively located/encoded on plasmids [i.e., *sed*, *selj*, *ser, ses*, and *set* (Bayles and Iandolo, 1989; Zhang et al., 1998; Omoe et al., 2003; Ono et al., 2008)] and on mutual relationships (i.e., two outbreak isolates and one non-outbreak isolate) were also prepared using the DNeasy Blood & Tissue kit (Qiagen, Hilden, Germany) and the Wizard gDNA Purification kit (Promega, Wisconsin, United States) (Table 2). These kits were selected based on their frequent use for WGS of *S. aureus* isolates (Gordon et al., 2014; Lee et al., 2015; Ekkelenkamp et al., 2018) and recommendations by leading institutes [EURL-CPS; (Merda et al., 2020)] in the field, respectively. The kits were used according to the manufacturer’s instructions and proposed protocols (Merda et al., 2020), respectively. Moreover, the GenElute Bacterial gDNA kit was tested on the same three isolates according to the specific protocol for Staphylococcal species, which requires the use of the expensive lysostaphin (Sigma-Aldrich, Missouri, United States) during bacterial cell lysis.

**TABLE 2:** Characteristics of selected DNA extraction kits.

DNA extraction kit	Price per sample* (€)	Average DNA conc. (ng/μL) ± s.d.	Average DNA yield (mg/ml starting material) ± s.d.	DNA purity (average ± s.d.)		Length range of DNA fragments (kb)	Remark
				A260/280	A260/230		
GenElute - NL GenElute	3.51 6.58	11.73 ± 2.23 38.02 ± 9.47	2.93 ± 0.56 9.50 ± 2.37	1.35 ± 0.02 1.82 ± 0.03	0.80 ± 0.08 1.87 ± 0.23	[55.78, >60.00] [30.18, >60.00]	Used by Belgian NRL-FBO (Nouws et al., 2020b)
DNeasy	3.77	4.45 ± 2.46	0.78 ± 0.43	1.68 ± 0.22	0.96 ± 0.29	[20.38, >60.00]	Frequently used for WGS of *S. aureus* (Ekkelenkamp et al., 2018)
Wizard	6.49	15.90 ± 11.11	1.59 ± 1.11	2.08 ± 0.16	2.44 ± 0.12	>60.00	Used by EURL-CPS (Merda et al., 2020)

The names of the DNA extraction kits are abbreviated, i.e., GenElute-NL: GenElute Bacterial gDNA kit using the protocol for Gram positive bacteria (without lysostaphin); GenElute: GenElute Bacterial gDNA kit using the protocol for Staphylococcal species (with lysostaphin); DNeasy: DNeasy Blood & Tissue kit; Wizard: Wizard gDNA Purification kit. For each of the used DNA extraction kits, averages and standard deviations (s.d.) of obtained DNA concentrations, yield, and purity were calculated from the three isolates (TIAC1798, TIAC1847, and TIAC3152). Fragment lengths are shown as ranges because the TapeStation Genomic DNA ScreenTape only gives exact measurements until 60 kb. *Prices as of February 2021 (excl. TVA, shipping, and handling costs). Prices were calculated from kits with highest throughput. With the exception of the cost of lysostaphin, the cost of extra products or materials required but not provided with the kit were not taken into account.

DNA concentration, purity, and integrity were determined with, respectively, the dsDNA HS and BR assay kits for the Qubit 4 fluorometer (Thermo Fisher Scientific, Schwerte, Germany), NanoDrop 2000 spectrophotometer (Thermo Fisher Scientific, Schwerte, Germany), and the Genomic DNA ScreenTape and Reagent kits for TapeStation 4200 electrophoresis (Agilent Technologies, Santa Clara, CA), according to the manufacturer’s recommendations.

### Library Preparation and Sequencing

One nanogram (in 5 μl) of each DNA extract was used for Nextera XT library preparation (Illumina, San Diego, CA). All libraries were sequenced on a MiSeq instrument (Illumina, San Diego, CA) using the MiSeq V3 chemistry to produce 2 × 250 bp paired-end reads, aiming at a theoretical sequencing depth of 60-fold.

### Whole Genome Sequencing Data Analysis

All bioinformatic analyses were performed using the respective tools on an in-house instance of Galaxy (Afgan et al., 2018). An online user-friendly Galaxy interface to perform raw read trimming, assembly and *se* gene detection (among other functionalities) using the bioinformatic methods and criteria as described in this manuscript is publicly available upon registration.^2^

#### Read Trimming and Assembly

The raw reads were trimmed using Trimmomatic 0.38 (Bolger et al., 2014) by removing Nextera XT adaptors and other Illumina-specific sequences (“Illuminaclip” set to value “NexteraPE-PE.fa:2:30:10”), removing low-quality residues at the start and end of the reads (“leading:10” and “trailing:10”), clipping reads when average Q-scores dropped below 20 over a sliding window of four residues (“slidingwindow:4:20”), and dropping reads shorter than 40 bases after processing (“minlen:40”). Trimmed reads were *de novo* assembled using SPAdes 3.13.0 (Bankevich et al., 2012) setting the “–careful” and “–cov-cutoff 10” options to reduce mismatches and short indels and remove low coverage contigs, respectively. Based on advice from a technical report by the ECDC, contigs below 1000 bp in length were removed using Seqtk seq 1.2^3^ using the “-L” option to improve assembly quality (ECDC, 2019). Relevant assembly statistics (N50, number of contigs, and median coverage against assembly) were calculated with Quast 4.4 (Gurevich et al., 2013) and are shown in Supplementary Table 3.

#### Database for *se* Gene Detection

The different publicly available databases for virulence gene detection in *S. aureus*, i.e., the Victors database (Sayers et al., 2019), VirulenceFinder database (Joensen et al., 2014), and VFDB_Full (Liu et al., 2019), were checked for the presence of *se* gene reference sequences. The VFDB_Full was selected because it was the most complete in containing reference sequences for the 27 *se* genes and was retrieved from its respective source^4^ on November 16, 2020. Some sequences were manually removed from and/or (re)added to the extended VFDB_Full database (i.e., *sep*, *ses*, *set*, *selw*, *selx*, *sey*, *selz*, *sel26*, and *sel27*; see Supplementary Data). Variants of *sec* (not separately annotated in the original VFDB_Full database) were also added so that variant subtyping of *sec* and *selu* (separately annotated in the original VFDB_Full database) was enabled. More information on sequences and accession numbers kept within (from the original VFDB_Full) and newly added to the extended VFDB_Full database is provided in Supplementary Table 4. The Supplementary Data also contains a FASTA file with sequences for all *se* genes present in the extended VFDB_Full database. Database sequences were clustered with an 85.0% sequence identity cutoff using the “cd-hit-est” function from CD-HIT 4.6.8 (Li and Godzik, 2006) to limit the detection of genes to one per cluster. This clustered database is also integrated in the online user-friendly interface for *se* gene detection.

#### Detection of *se* Genes Using the Extended Virulence Factor Database_Full Database

All samples were genotypically characterized for the presence of *se* genes, using two methods: (i) Aligning assemblies with BLAST + 2.6.0 (Camacho et al., 2009) and (ii) mapping trimmed reads with SRST2 0.2.0 (Inouye et al., 2014) with the options “–max-divergence 10,” “–min-coverage 60,” “–gene-max-mismatch 10,” and “–max-unaligned-overlap 150,” both against the extended VFDB_Full database. For the isolate with BioSample accession SAMN13134218, only the assembly was publicly available, limiting *se* gene detection to the assembly based approach using BLAST+ (i.e., no gene detection with SRST2 could be performed). Hits identified with <60.0% query coverage and/or >10.0% sequence divergence for SRST2 or with <60.0% query coverage and/or <90.0% sequence identity for BLAST+ were omitted. The best hit for each detected database cluster for BLAST+ was determined based on a previously described allele scoring method (Larsen et al., 2012). In the case of unexpected results, manual contig alignment against the corresponding reference gene using BLAST (Altschul et al., 1990) was performed to identify assembly artifacts. A gene was considered present with WGS when it could be detected with SRST2 and/or BLAST+.

#### Phylogenetic Analysis of the *S. aureus* Isolates Using Core Genome Multilocus Sequence Typing-Typing

*In silico* cgMLST-typing was performed as previously described (Bogaerts et al., 2021) by aligning assemblies using BLAST+ against the *S. aureus* cgMLST scheme of PubMLST (Jolley et al., 2018) containing 2208 loci (downloaded on January 3, 2021). Only exact allele calls (i.e., requiring a full-length, 100.0% identical match) were accepted. For tree construction, loci called in <80.0% of samples were stripped from the allele call matrix. A minimum spanning tree based on the allele call matrix was created using GrapeTree 1.5.0 (Zhou et al., 2018) with the “method” option set to “MSTreeV2′,” and afterward visualized using FigTree 1.4.4 (Rambaut, 2007).

### Identifying the Completeness of *se* Gene Profiling and Evaluating the Performance of Whole Genome Sequencing in Inferring Phylogenetic Relationships

To evaluate if WGS can identify the complete *se* gene profile of *S. aureus* isolates, the *se* gene profiles determined with SRST2 and BLAST+ against the extended VFDB_Full database were compared with the expected profiles obtained using *in silico* PCR. From this comparison, WGS-based *se* gene detection results were classified as either true positives (TPs), false positives (FPs), true negatives (TNs), or false negatives (FNs), for which definitions are shown in Supplementary Table 5.

Moreover, phylogenetic relationships inferred from the cgMLST profiles determined with WGS were compared to the *a priori* known relationships to evaluate the additional benefit of WGS in SFP investigation as a proof of concept.

The potential influences of the tested DNA extraction kits and protocols on both *se* gene detection and inferring phylogenetic relationships were assessed similarly.

## Results

### Selected Isolates and Their Characteristics

To identify the benefits of WGS in SFP investigation, 13 in-house sequenced isolates (Table 1) were selected based on *a priori* known relationships to include outbreak (n: 3) and non-outbreak (n: 10) isolates and on the previously assessed presence of *se* and/or SE (i.e., *sea* to *see*, *seg* to *selj*, *sep*, and *ser*, and/or SEA to SEE). The selection of isolates included isolates of food (n: 7) and human (n: 6) origin, including two (TIAC1993 and TIAC1994) that were isolated from nose and throat of one human case. Because the previously identified *se*/SE profiles of the in-house sequenced isolates was limited to a maximum 11 out of 27 described *se* genes, the complete *se* gene profile per isolate was extended with *in silico* PCR (Supplementary Table 6). To cover the complete repertoire of currently described *se* genes (*ses*, *set*, *selv*, *sey*, *sel26*, and *sel27* that were missing in the in-house sequenced isolates), a publicly available WGS data of three isolates were added (Table 1). The *in silico* PCR was similarly applied for the publicly available WGS data to confirm the previously reported *se* genes (Fursova et al., 2020; Merda et al., 2020). Based on the investigated specificity of all primer sets published across the literature (see Supplementary Data and Supplementary Table 1), 139 out of 155 primer pairs (i.e., 89.7%) were selected for the *in silico* PCR. From the *in silico* PCR results, seven sets (i.e., 5.0% of the 139 used sets) were, moreover, identified to not consistently detect their target genes even when present (see Supplementary Data and Supplementary Table 6). This is most likely because these primers were not designed to anneal in sufficiently conserved gene regions. More information on the *in silico* PCR can be found in the Supplementary Data and Supplementary Table 6. For *se* genes or SEs whose presence was identified with conventional methods (EURL-CPS multiplex PCR assays and/or VIDAS/SET-RPLA), results of the *in silico* PCR were fully congruent (Table 1). For all *se* genes that were previously reported to be present in the publicly available WGS data [see Table 1 and Supplementary Table 2 (Fursova et al., 2020; Merda et al., 2020)], their presence was accordingly identified with *in silico* PCR except for *selv* that was not detected in SAMN13134218 based on the *in silico* PCR (see Supplementary Table 6 and Table 1) although it was previously reported to be present (Fursova et al., 2020). Moreover, across these three isolates analyzed with public data, *in silico* PCR enabled the detection of three additional *se* genes (i.e., *sem* in SAMN02391177, *selx* in SAMN02403200 and SAMN13134218, and *selw* in the three isolates; Supplementary Table 6 and Table 1), previously not identified to be present (Fursova et al., 2020; Merda et al., 2020).

### Identifying the Completeness of Whole Genome Sequencing-Based *se* Gene Profiling

By comparison with the expected *se* gene profiles (Table 1), it was assessed whether WGS could identify the complete *se* gene repertoire (reportable range) of *S. aureus* isolates using the extended VFDB_Full database (Table 3). The presence or absence of, in total, 27 different *se* genes, among which are *sec* and *selu* variants, and of two pseudogenes was determined. Compared with solely using the VFDB_Full database, the presence or absence of eight extra genes (*ses*, *set*, *selw* to *selz*, *sel26*, and *sel27*) and *sec* variants could, hence, be determined.

**TABLE 3:** WGS-based *se* gene profile of the selected in-house isolates and influence of the DNA extraction kits on se gene profiling.

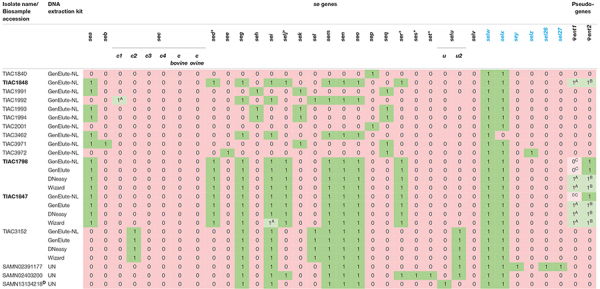

For each isolate, the *se* gene profile determined with WGS based on the extended VFDB_Full database is indicated. Isolates originating from the same outbreak are shown in bold. Gene presence is indicated as “1” in a green box, absence as “0” in a pink box. When a gene is detected only by SRST2 or BLAST+, it is shown with “1” in a light green box. When a gene was missed compared to the *in silico* PCR results (Table 1), it is indicated with “0” in a light pink box. Gene names with an asterisk “*” are genes exclusively encoded on plasmids. Gene names indicated in blue are genes whose presence/absence could solely be identified by using the extended VFDB_Full database (because these genes were absent in the original VFDB_Full database). Enterotoxin genes whose variants were determined with the extended VFDB_Full database, are shown. For the three isolates that were tested with the different DNA extraction kits, the *se* gene profiles are also shown. The names of the DNA extraction kits are abbreviated, i.e., GenElute-NL: GenElute Bacterial gDNA kit using the protocol for Gram positive bacteria (without lysostaphin); GenElute: GenElute Bacterial gDNA kit using the protocol for Staphylococcal species (with lysostaphin); DNeasy: DNeasy Blood & Tissue kit; Wizard: Wizard gDNA Purification kit; UN: unknown.

*^*A*^Gene not detected with BLAST+.*

*^*B*^Gene not detected with SRST2.*

*^*C*^Discrepant result for the pseudogenes, compared to the expected *se* gene profile as determined with *in silico* PCR (Table 1).*

*^*D*^Only the assembly was publicly available to use for *se* gene detection in this study. Therefore, only BLAST+ could be used.*

For *se* gene detection, the WGS-determined profile per isolate (Table 3) was identical to what was expected (i.e., based on the conventional methods and *in silico* PCR as indicated in Table 1). Indeed, across the, in total, 432 observations (27 *se* genes for each of the 16 isolates), i.e., 127 positive and 305 negative observations, there were no FPs or FNs. In comparison with the analyses performed during the initial SFP outbreak investigation [Table 1; (Denayer et al., 2017)], WGS provided information on the presence/absence of 17 extra *se* genes and additionally allowed subtyping of *sec* and *selu* variants (Table 3). For the isolates for which public WGS data were available (Fursova et al., 2020; Merda et al., 2020), the WGS-based *se* gene detection methods applied in this study using the extended VFDB_Full database enabled the detection of three extra *se* genes (i.e., *sem* in SAMN02391177, *selx* in SAMN02403200 and SAMN13134218, and *selw* in the three isolates) in line with the expected characteristics within Table 1. The *selv* gene that was previously detected in SAMN13134218 (Fursova et al., 2020) was missed with the WGS method using the extended VFDB_Full database in this study. However, manual alignment of the contigs with the NCBI reference sequence of *selv* (Genbank reference EF030427.1) with BLAST confirmed its absence as was similarly found with the *in silico* PCR and, hence, the results of this study. Between the *se* gene profiles obtained with SRST2 and with BLAST+, only one difference was observed for *se* gene detection, related to a fragmentation of the assembly in the *sec* gene for TIAC1992 that led to its missed detection only with BLAST+. By using the extended VFDB_Full database, WGS, thus, enabled complete *se* gene profiling using both the read mapping-based (SRST2) and assembly based (BLAST+) approach.

For the WGS-based detection of the Ψ*ent* pseudogenes, there were some discrepancies in isolates TIAC1798, TIAC1847, and TIAC1848 compared with what was expected (cfr. Table 1). One of both pseudogenes was systematically missed with BLAST+ and SRST2, which could be explained by the clustering of both genes in the database that limits their detection to only one of them. Differences in the detected Ψ*ent* pseudogene in TIAC1848 by BLAST+ and SRST2 can be explained by the fact that both methods use different allele scoring methods. However, manual alignment of the respective contigs with a Ψ*ent1-2* reference sequence (Genbank reference MN450303.1) confirmed the presence of both pseudogenes in the TIAC1798, TIAC1847, and TIAC1848 isolates with 100.0% sequence identity and query coverage (data not shown). Different DNA extraction kits and protocols (i.e., including lysostaphin in the bacterial lysis step) were tested for their influence on detecting *se* genes. Although lysostaphin increased the DNA yield, sufficient amounts and concentrations were obtained for Nextera XT library preparation without its usage [Table 2; (Illumina, 2018)]. For the detection of the 27 *se* genes, identical profiles were obtained with WGS for all samples (i.e., WGS data of an isolate processed with different DNA extraction kits) per isolate, irrespective of the DNA extraction kit, when compared with the expected *se* gene profiles. No problems in detecting any of the exclusively plasmid-encoded *se* genes (i.e., *sed*, *selj*, *ser, ses*, and *set*) were observed. When comparing *se* gene profiles obtained with SRST2 and BLAST+ separately for the samples per isolate, only one difference was obtained, related to the missed assembly-based detection of *sei* due to assembly fragmentation in TIAC1847 processed by the Wizard gDNA Purification kit. Therefore, no influence of the kit on the detection of *se* genes, whether or not plasmid-encoded, could be identified. Difficulties with the detection of both Ψ*ent* pseudogenes could again be observed across all samples of TIAC1798 and TIAC1847 with both detection methods, explained by the clustering of the pseudogenes in the database in combination with differences in the allele scoring methods of BLAST+ and SRST2. Manual alignment of the contigs against the Ψ*ent1-2* reference (Genbank reference MN450303.1), confirmed the presence of both pseudogenes with 100.0% sequence identity and query coverage in both isolates for the different extraction kits (data not shown).

### Performance Evaluation of Whole Genome Sequencing-Based Isolate Relatedness Determination

**FIGURE 1:**
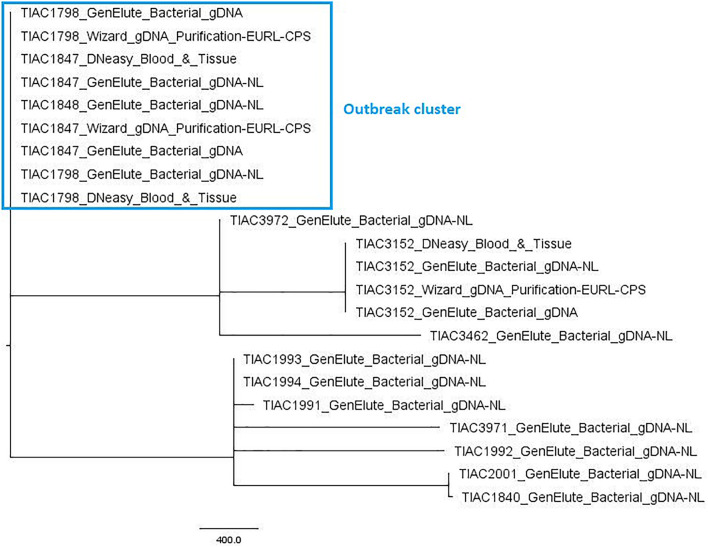
Minimum spanning tree based on cgMLST for *S. aureus* isolates. A minimum spanning tree was created with GrapeTree using the MSTreeV2 method on all in-house sequenced outbreak and non-outbreak samples, i.e., all isolates, some of which processed with different DNA extraction kits. The sample names in the figure consist of the name of the respective isolate and an abbreviation of the applied DNA extraction kit, i.e., GenElute_Bacterial_gDNA-NL: GenElute Bacterial gDNA kit using the protocol for Gram-positive bacteria [without (i.e., no) lysostaphin, NL]; GenElute_Bacterial_gDNA: GenElute Bacterial gDNA kit using the protocol for Staphylococcal species (with lysostaphin); DNeasy_Blood_&_Tissue: DNeasy Blood & Tissue kit; Wizard_gDNA_Purification: Wizard gDNA Purification kit. The outbreak samples (outlined in the blue box) from food origin (TIAC1798) consistently cluster together with those of human cases (TIAC1847 and TIAC1848) while non-outbreak samples (TIAC1840, TIAC1991, TIAC1992, TIAC1993, TIAC1994, TIAC2001, TIAC3152, TIAC3462, TIAC3971, and TIAC3972) are separated from the outbreak cluster and delineated per isolate (except for TIAC1993 and TIAC1994 sampled from the same person that also cluster together because of their identical cgMLST profiles). The scale bar represents the number of cgMLST allele differences between samples. No cgMLST allele differences were identified between the outbreak isolates, nor isolates processed with different DNA extraction kits.

The performance of WGS in isolate relatedness determination was evaluated through comparison of inferred phylogenetic relationships by cgMLST-typing with the *a priori* known relationships of the in-house sequenced isolates. Of all 2208 core gene loci, on average, 91.2 ± 5.7% could be detected with 100.0% query coverage and sequence identity across all isolates. Figure 1 visualizes the relatedness between the *S. aureus* isolates, based on cgMLST. The tree demonstrates that the outbreak strain isolated from the food matrix (TIAC1798) clustered together with those of the human cases (TIAC1847 and TIAC1848) in a single clade carried by one branch, being separated from all other non-outbreak isolates. Similarly, both strains isolated from an identical human case at different locations (i.e., TIAC1993 and TIAC1994) also clustered together on one branch. No cgMLST allele differences were identified between any of the outbreak isolates within the outbreak clade or between the two isolates from the same human case. WGS-based cgMLST analyses, therefore, provided inferred phylogenetic relationships between the *S. aureus* isolates that were in accordance with the *a priori* known relationships.

Moreover, the used DNA extraction kit and workflow had no influence on the obtained cgMLST profiles or on the retrieved relationships between isolates. Indeed, no cgMLST allele differences were obtained between samples from the same isolate and, thus, clustered together per isolate in one single clade carried by a single branch, irrespective of the applied DNA extraction kit.

## Discussion

The goal of this study was to assess the potential benefits of WGS compared with conventional molecular methods currently used in the investigation of SFP outbreaks. For this purpose, WGS was employed for *se* gene detection applying a custom database, and its profiles were compared with those previously obtained by routine methods (the EURL-CPS multiplex PCR and/or SET-RPLA/VIDAS analyses) and extended by *in silico* PCR. To cover the complete arsenal of currently described *se* genes, we included publicly available WGS data (Fursova et al., 2020; Merda et al., 2020) that were also verified with *in silico* PCR. Moreover, the performance of WGS-based isolate relatedness determination was evaluated using cgMLST on a data set with *a priori* known phylogenetic relationships. Within the scope of SFP investigation, different DNA extraction kits were tested for their influence on the comparability of WGS data.

This study demonstrates that WGS presents a valid alternative to molecular methods, serving as the ultimate multiplexing approach for *se* gene detection in *S. aureus* isolates. WGS enabled complete *se* gene profiling within one single assay. In contrast to WGS, PCR-based methods for the analysis of *se* gene profiles tend to miss the detection of genes that were not targeted by the approach, or when using primer pairs not targeting sufficiently conserved gene regions (see Supplementary Table 1). As a result, SFP outbreaks caused by non-classical enterotoxins that cannot be detected with existing commercial kits (i.e., SEs other than SEA to SEE) risk being left unresolved when also not targeted with PCR. However, complete PCR-based *se* gene profiling is very time-consuming and labor-intensive. Besides giving a first indication on the possible causality of a strain toward SFP outbreaks, knowledge on the complete *se* gene profile of isolates is also important in SFP surveillance. Its combination with clinical data of human cases can help in risk assessment of *S. aureus* strains to predict the potential pathogenicity of isolates. By analyzing isolated strains of SFP outbreaks for their complete *se* gene profile, more knowledge can moreover be acquired on *se* genes most commonly involved in SFP outbreaks.

Both SRST2 and BLAST+ showed highly similar WGS-based *se* gene detection outputs. Across all isolates, there was only one *se* gene left undetected because of assembly fragmentation when using BLAST+. The application of SRST2 read mapping is, hence, preferred for WGS-based *se* gene profiling. Although not encountered in this study, assembly issues might not only result in BLAST+ being more prone to the missed detection of *se* genes, but also the false positive detection of (pseudo)genes (Mason et al., 2018) that have recombined to new *se* genes. Indeed, *selv* and *selu* genes have been described to be formed from recombination events in *sem* and *sei* genes, or Ψ*ent1* and Ψ*ent2* pseudogenes, respectively (Thomas et al., 2006). More extensive performance evaluation of applying BLAST+ and SRST2 for *se* gene detection, which was, however, not the main goal of this study, would require a higher number of *S. aureus* isolates to be analyzed. Although previous research shows performance differences to be limited for gene detection in other bacterial species when using high-coverage data sets (Bogaerts et al., 2021), it is an interesting future research topic to also verify for *S. aureus* enterotoxin gene detection.

Whole genome sequencing also allows subtyping of *se* variants. Recently, an increasing number of SE variants are being described (Blaiotta et al., 2004; Fernández et al., 2006; Kohler et al., 2012; Johler et al., 2016; Aziz et al., 2020; Etter et al., 2020; Merda et al., 2020). Multiple variants (such as for SEC) are already shown to exhibit different structural and superantigenic features (Etter et al., 2020), but the influence on their emetic activity has not yet been investigated. However, in the future, based on this knowledge, subtyping of variants can become increasingly important in predicting the emetic potential of *S. aureus* strains. In this study, subtyping of *sec* and *selu* was done as proof of concept, but the applied *se* gene detection thresholds (i.e., >60.0% query coverage and >90.0% sequence identity for BLAST+ and >60.0% query coverage and <10.0% sequence divergence for SRST2) allow also detecting other *se* variants, including novel ones, without subtyping. However, WGS offers the potential to expand *se* subtyping to all variants when annotated as such in the applied gene detection database. This is an additional added value of WGS compared with conventional PCR-based methods, in which extra Sanger sequencing analyses of the obtained amplicons are often required for further subtype identification (Collery and Smyth, 2007).

For WGS to provide full *se* gene profiling, the application of a database containing reference sequences for all *se* genes and, if subtyping is of interest, also all variants is indispensable. However, publicly available reference databases for virulence gene detection in *S. aureus* isolates (Joensen et al., 2014; Liu et al., 2019; Sayers et al., 2019; Merda et al., 2020) do not enable complete *se* gene profiling (e.g., *ses, set*, *selw* to *selz*, *sel26* and *sel27* are missing in the VFDB_Full database, and *ses, set*, *selv* to *selz*, *sel26* and *sel27* in the VirulenceFinder DataBase) or subtyping of *se* variants (because they are not or ambiguously annotated in the databases). Studies using these databases as such (Huang et al., 2017; Petit and Read, 2018; Fursova et al., 2020; Karki et al., 2020; Merda et al., 2020; Schwendimann et al., 2021), thus risk underestimating the prevalence of *se* genes, potentially affecting obtained results. Indeed, more *se* genes were reported to be present in the isolates with publicly available WGS data after reanalysis with our methods compared with their previously WGS-determined *se* gene profiles, mainly because of the lack of complete databases in the other studies (Fursova et al., 2020; Merda et al., 2020). Recently, the EURL-CPS developed a workflow for *S. aureus* analysis in the scope of SFP investigation, i.e., NAuRA, using a database that allows almost complete (*selw* is missing and no variant subtyping) *se* gene detection (Merda et al., 2020). NAuRA is available on github, and the Uniprot accession numbers of SE protein sequences used as reference in their genomic analyses are available in the supplementary data of the corresponding publication (Merda et al., 2020). NAuRA can be locally installed by an experienced bioinformatician, who might not always be available in each NRL. Nevertheless, the implementation of a publicly available database that is easily accessible and queryable is favorable in a routine setting because it supports the comparability of WGS data results, especially important during SFP investigation. To make WGS more approachable, it is, thus, important that reference databases enabling full *se* gene profiling are made publicly available through online open repositories. Moreover, these databases should be continuously updated with the state-of-the-art knowledge so that full *se* gene profiling and variant subtyping is possible. A FASTA file containing the *se* gene sequences and their corresponding accession numbers used for the database constructed in this manuscript was added to the Supplementary Data. This database was also integrated in the open access online interface^5^ (registration required), using the parameters as described in this manuscript, to moreover allow user-friendly *se* gene detection.

Although present in the extended VFDB_Full database, in our study, discrepant observations were identified with the detection of Ψ*ent* pseudogenes when using WGS. This was related to (i) database sequence clustering, which limits gene detection to one per cluster, and (ii) differences in the allele scoring methods of SRST2 and BLAST+ that decide upon the detected gene (allele) per cluster. Because both Ψ*ent* pseudogenes coexist on the enterotoxin gene cluster (*egc*) (Jarraud et al., 2001), one of them risks being left undetected when using BLAST+ or SRST2. However, because pseudogenes are non-functional (Letertre et al., 2003b; Liang et al., 2016), the impact of these discrepancies can be regarded as negligible. Nevertheless, if their detection would be of special interest, we suggest performing manual alignment of the assembly with a Ψ*ent1-2* reference sequence to identify their potential joint presence as performed in this study. This extra alignment step still remains much less time-consuming compared with the Sanger sequencing analyses that would be required when using conventional PCR methods to distinguish pseudogene presence from those of the *selu* gene (Collery and Smyth, 2007). Sequence clustering of gene variants is, however, important when applying large databases, such as the VFDB_Full, to aid in identifying the most appropriate gene (allele) within the cluster. Without clustering of gene variants in combination with default gene detection thresholds, a hit to all of those variants might be picked up if the gene is present, whereas manual inspection would reveal that it concerns only one gene (variant) that is present. Based on sequence similarity between all *se* genes and variants (Merda et al., 2020), we decided to apply a sequence identity threshold of 85.0% to cluster the extended VFDB_Full database, combined with default gene detection thresholds (i.e., >60.0% query coverage and >90.0% sequence identity for BLAST+ and >60.0% query coverage and <10.0% sequence divergence for SRST2). Most *se* genes and variants are consequently clustered together per gene except for *selu* and the pseudogenes or *sem* and *selv* that are present in the same cluster together. With the combination of both clustering and gene detection thresholds, a sufficient level of sensitivity and specificity is acquired to detect novel *se* variants and avoid FP and FN gene detection, respectively. The importance of appropriate gene detection (and database sequence clustering) thresholds is also shown by analyzing publicly available WGS data. Based on *in silico* PCR and manual inspection of the contigs, the *selv* gene was found to have been previously falsely detected within these public WGS data [SAMN13134218; (Fursova et al., 2020)]. This could likely be explained by the application of default SRST2 gene detection thresholds (i.e., >90.0% query coverage and <10.0% sequence divergence), which are not stringent enough to distinguish between the present *sem* and *sei*, and the absent *selv*, in the absence of a database clustering step. Indeed, with these detection criteria, *sem*, *sei*, and *selv* were detected in the previous study (Fursova et al., 2020) although only *sem* and *sei* were determined to be present here. Although the gene detection criteria in our study were looser, the presence and absence of these genes could be correctly identified because they were combined with a database whose sequences were clustered. Of all 27 enterotoxins described to date, *selv* was, thus, determined not to be present in any of the selected strains/public data. Expanding the number of isolates in this study would likely also increase the potential of covering *selv*. However, because this gene is not targeted by the VIDAS/SET-RPLA immunoassays or EURL-CPS multiplex PCR applied routinely in-house and is found to be only scarcely prevalent (Merda et al., 2020), including an isolate containing *selv* would not be straightforward. Moreover, because *sem* and *selv* are not yet found to co-occur within the same strain to date (Thomas et al., 2006; Argudín et al., 2010), detection issues related to their clustering are not expected if an isolate containing *selv* would have been added to the study (as was attempted with the public WGS data). Nor are *selu* and pseudogene detection issues expected related to their clustering in the database. When adding more variant sequences to the database, as elaborated above to allow subtyping, it is necessary that applied gene detection thresholds and database sequence identity thresholds for clustering are further reviewed to ensure accurate and specific *se* variant detection.

To examine the completeness of WGS-based *se* gene profiling in *S. aureus* isolates, previously assessed *se* gene profiles were extended with *in silico* PCR. Using *in silico* PCR to validate the detection of *se* genes in all in-house sequenced isolates and isolates for which WGS data were publicly available might seem conflicting because both approaches are dependent on the same WGS data. However, although BLAST+ and SRST2 approaches detect complete *se* gene coding sequences, *in silico* PCR only evaluates primer binding sites and obtained amplicon sizes. Sixteen out of 155 primer pairs (i.e., 10.3%) described in literature did not result in a specific *in silico* product. Moreover, from the 139 primer sets used for the *in silico* PCR, another seven pairs (i.e., 5.0%) were identified to anneal in non-conserved gene regions leading to the missed detection of its corresponding target gene in some isolates tested within this study (see Supplementary Data). Increasing the number of *S. aureus* isolates, likely also supplements the gene sequence variability and potentially leads to the identification of extra primers that are not developed in sufficiently conserved regions. Although this remains an interesting research question for further research, with the limited number of isolates present, we highlight the added value of incorporating genomic data for more in-depth primer optimization (Vanneste et al., 2018) and show the importance of profound sensitivity and specificity studies for developed primer sets before publication. Indeed, if these primer pairs were used in a conventional PCR, a FN result might have been obtained, impeding a full correct characterization of the *se* profile. By applying the *in silico* PCR approach with 139 PCR primer pairs (i.e., 89.7%) selected based on their specificity, something that would be less feasible in a conventional approach, this risk was circumvented and even showed an added value. For the 11 conventionally PCR tested *se* genes, all results were completely congruent with the *in silico* PCR results, thereby validating our approach. Moreover, the *in silico* PCR indicated an inconsistency with the reported *se* profiles in a previous study, which was further confirmed using manual alignments and explained by the applied computational methods. Our review of published primer pairs and *in silico* investigation for their specificity can, therefore, also be valuable information for laboratories not having the resources to perform WGS.

Besides the fact that complete *se* gene profiling can provide an initial insight into the potential causality of strains toward outbreaks, WGS also allows inferring more refined phylogenetic relationships between *S. aureus* isolates based on cgMLST-typing. Because 91.2 ± 5.7% of the 2208 core genome loci could be typed, a relatively high number of genomic markers was available to reliably resolve isolate relationships in this study. cgMLST-typing has gained interest with regard to standardization and harmonization because of the transferability of the applied scheme, enabling the use of the same database (Sabat et al., 2017), which allows SFP outbreaks to be investigated across laboratories. Although other cgMLST schemes have been developed by multiple instances (Leopold et al., 2014), we adopted the PubMLST scheme to infer *S. aureus* isolate relationships in this study because it is the most frequently used scheme, that is, moreover, also applied by the EURL-CPS (Merda et al., 2020). The ultimate level of discriminative power is provided by SNP analysis. However, this can only be used to further fine-tune relationships between closely related strains. Because the overall distance based on cgMLST loci differences between the isolates in this study is large, except for the outbreak strains and two identical strains TIAC1993 and TIAC1994 isolated from one human case, picking a suitable reference genome for SNP analysis would be impossible. Increasing the number of outbreak and non-outbreak isolates can be used to illustrate the discriminative power of WGS even more than in this study. However, the rapid course of disease and the complexity of *S. aureus* isolation because of potentially affected strain viability, often complicate the collection of strains during outbreak investigation. Moreover, not many studies were found to analyze *S. aureus* isolates with WGS in the scope of SFP outbreak investigation. Our study, by demonstrating its added value, might contribute in stimulating the use of WGS in SFP outbreak investigations so that its benefits can be fully exploited.

Data sharing between laboratories is crucial in the investigation of SFP outbreaks. However, the comparability of WGS data can potentially be affected by impaired plasmid extraction performances of commercial DNA preparation kits (Becker et al., 2016; Nouws et al., 2020b), especially when harboring important virulence genes, such as *se* genes. Therefore, we tested a frequently used kit (DNeasy Blood & Tissue kit), the kit recommended by the EURL-CPS for DNA preparation of *S. aureus* isolates (Wizard gDNA Purification kit) and the kit used at the Belgian NRL-FBO/NRL-CPS (GenElute Bacterial gDNA kit) for their influence on WGS data analyses of *S. aureus*. Although the DNeasy Blood & Tissue and Wizard gDNA Purification kits were previously described to have impaired plasmid extraction performances in Gram-negative species, possibly leading to missed WGS-based detection of plasmid-encoded genes (Becker et al., 2016; Pasquali et al., 2019; Nouws et al., 2020b), this assumption could not be extrapolated to *S. aureus* isolates based on the results in our study. Indeed, identical profiles of detected *se* genes, whether or not plasmid-encoded, were obtained between the samples per isolate, irrespective of the applied DNA extraction kit. Moreover, the use of different kits had no influence on inferred cgMLST-based isolate relationships. Kit choice to prepare DNA of *S. aureus* for WGS can, thus, be based on other factors. Because of its earlier communication to be appropriate for WGS data analysis of Gram-negative and -positive bacteria (Nouws et al., 2020b), the GenElute Bacterial gDNA kit can be of interest for usage in laboratories, such as NRLs, investigating multiple foodborne pathogens. We moreover examined the necessity of employing the expensive lysostaphin enzyme within its protocol as specified for Staphylococcal species (Zhao et al., 2012). Because sufficient amounts of DNA were obtained without using lysostaphin, and its use did not influence the outcome of WGS analyses, the GenElute Bacterial gDNA protocol for Gram-positive bacteria not using lysostaphin is especially beneficial for routine application because discarding lysostaphin nearly halves the cost per sample (Table 2).

Although a limited number of isolates was used in this study, it is clear that WGS has benefits in the investigation of SFP outbreaks, yielding information on the complete *se* profile and relatedness between strains, all within one single test. Although WGS can provide, besides *se* gene detection, complete isolate characterization by detecting other virulence and antimicrobial resistance (AMR) genes, such as, among others, the virulence *tsst* gene (Kulhankova et al., 2014) responsible for, e.g., menstrual toxic shock syndrome, or the AMR *mecA* gene causing methicillin resistance (Peacock and Paterson, 2015), these characteristics were not addressed in this study because of their irrelevance in SFP or its treatment (Fisher et al., 2018), and because no conventional metadata concerning these characteristics were available with which to compare the performance of WGS-based detection. Performing *in silico* PCR to obtain information on the presence/absence of these genes would be virtually impossible because of the large numbers of AMR and virulence genes that exist. However, other studies have already demonstrated that WGS scores very well in predicting the AMR phenotype within *S. aureus* (Gordon et al., 2014; Cunningham et al., 2020). For WGS to yield all benefits in SFP investigation, it is important that *S. aureus* strain isolation is successful. Because the viability of *S. aureus* can be affected by food processing through, e.g., heating, while integrally preserving the emetic properties of the produced enterotoxins (Fisher et al., 2018), a proportion of the suspected foods might be left unable to be investigated with the proposed method. Metagenomics sequencing of the complete sample without strain isolation might offer a solution relevant for further research (Boers et al., 2019). However, both WGS and metagenomics sequencing only deliver information at the genotypic level. Ideally, sequencing-based methods should be used in first line to screen for *se* gene presence and can then be combined with a method that allows detection of all produced SEs at the protein level. Nevertheless, through complete *se* gene detection, WGS enables broadening the insight in *S. aureus* and SFP and examines the potential of isolates to produce SEs and to play a role in SFP. Moreover, thanks to its ultimate discriminatory power, WGS can simultaneously more accurately pinpoint strains as the cause for a SFP outbreak and, thus, accelerate its management. Conclusively, this study shows the added value of using WGS in SFP outbreak investigation and encourages its use in a routine setting.

## Data Availability Statement

The datasets generated for this study can be found in the NCBI SRA repository under the accession number PRJNA750393 (http://www.ncbi.nlm.nih.gov/bioproject/750393). Corresponding ac-cession numbers are listed in Supplementary Table 7.

## Author Contributions

SN, NR, and SDK conceived and designed the study. SDK supervised the project. SN performed the wet lab experiments. BB and KV provided the bioinformatic tools in Galaxy. SN and BB performed the bioinformatic analysis. SD and BV were responsible for the former generation of all results obtained with the conventional methods. SN and SDK participated in the interpretation of the results and wrote the manuscript. KV, BB, LL, NR, and KM provided specialized feedback on the obtained results. All co-authors commented and approved the submitted version.

## Conflict of Interest

The authors declare that the research was conducted in the absence of any commercial or financial relationships that could be construed as a potential conflict of interest.

## Publisher’s Note

All claims expressed in this article are solely those of the authors and do not necessarily represent those of their affiliated organizations, or those of the publisher, the editors and the reviewers. Any product that may be evaluated in this article, or claim that may be made by its manufacturer, is not guaranteed or endorsed by the publisher.

## Footnotes

1
https://www.ncbi.nlm.nih.gov/tools/primer-blast/index.cgi

2
https://galaxy.sciensano.be/tool_runner?tool_id=pipeline_staphylococcus

3
https://github.com/lh3/seqtk

4
http://www.mgc.ac.cn/VFs/download.htm

5
https://galaxy.sciensano.be/tool_runner?tool_id=pipeline_staphylococcus
