# Supplementary material for: Whole Genome Sequencing Provides an Added Value to the Investigation of Staphylococcal Food Poisoning Outbreaks
Source: Front Microbiol. 2021 Nov 2;12:750278. doi: 10.3389/fmicb.2021.750278 (PMC8593433; doi:10.3389/fmicb.2021.750278)
Supplement: Supplementary file 1 [file Data_Sheet_1.docx]

Supplementary Material

# Supplementary Data

## Investigation of specificity of conventional PCR primer pairs

For the *in silico* PCR, a literature study was performed to collect all published conventional PCR primer sequences (Supplementary Table S1). Since no *selv* specific primer pairs for PCR were described in literature, cloning primers described in literature were used for *selv* (by removing the 5’ end restriction sites underlined in the corresponding sequences) (Supplementary Table S1).

All primers were *in silico* investigated for their specificity to the target gene (and, if not yet known, their amplicon size) by aligning the primer pairs against the NCBI nucleotide collection for *S. aureus* (taxid: 1280) using Primer-BLAST. Besides the criteria previously described (Vanneste et al., 2018), default parameters were applied that assessed whether a primer pair allowed the formation of an *in silico* amplicon. A primer pair was determined to be specific when it allowed *in silico* detection of its target gene with the expected amplicon size, and without aspecific detection of another Staphylococcal gene. When the obtained amplicon size for some primer pairs was (slightly) different compared to the one that was indicated in the respective study, it was indicated in the Supplementary Table S1. This difference in amplicon size was overall very limited, being lower than the variation (15%) that is accepted by sensitive electrophoresis methods determining amplicon size (Agilent Technologies, 2015).

89.03% or 138 of 155 primer sets specifically detected the target gene without annealing to another Staphylococcal gene (with similar amplicon size). Thirteen of 17 aspecific primer pairs (shown in grey and light green in Supplementary Table S1) targeted non-conservative *se* gene regions, leading to (i) False positive detection of *se* genes described in literature to be highly similar in their nucleotide sequence (n: 9); or (ii) False positive detection of other Staphylococcal (*se*) genes without demonstrated sequence similarity (n: 4). The remaining four aspecific primer pairs were determined to not target any Staphylococcal gene according to the preset criteria.

Because conventional PCR detection does not enable the discrimination between *selu*, *selu2* and pseudogenes *Ψent1* and *Ψent2* due to high sequence similarity (Heymans et al., 2010; Liang et al., 2016), their detection is generally followed by DNA Sanger sequencing for confirmation (Collery and Smyth, 2007). In other words, no primer pairs were found in literature that enabled detection of *selu* and *selu2* without aspecifically detecting their precursor pseudogenes, i.e. *Ψent1-2*. Therefore, one of the aspecific primer pairs was selected for the *in silico* PCR of *selu* or the pseudogenes (shown in green in Supplementary Table S1; primer set selu-1). In total 139 primer pairs were thus used for the *in silico* PCR (i.e. 89.68%). When the primer pairs indicated the presence of *selu*, *selu2*, or *Ψent1-2*, *in silico* PCR was repeated using a primer pair (forward: 5’- TGA TAA TTA GTT TTA ACA CTA AAA TGC G-3’; reverse: 5’- CGT CTA ATT GCC ACG TTA TAT CAG T-3’; (Letertre et al., 2003)) targeting the complete gene length. The amplicon sequence was extracted and aligned against those of NCBI sequences (see Supplementary Fig. S1) harbouring *selu* (Genbank reference AY158703.1), *selu2* (Genbank reference MN450302.1), and *Ψent1-2* (Genbank reference MN450303.1) using CLC Sequence Viewer 8.0 with the aim to type the specific gene based on similarity with the reference sequences.

## *In silico* PCR to determine the *se* gene profile of the isolates

The 139 primer pairs were used in addition to conventional methods to extend *se* gene profiling to its complete arsenal, and to verify the previously reported *se* profile obtained from WGS data (Fursova et al., 2020; Merda et al., 2020). For the *in silico* PCR, these 139 primer pairs were aligned against the assemblies of the 13 in-house sequenced *S. aureus* isolates processed with the GenElute Bacterial gDNA kit, and the downloaded assemblies of the three isolates for which WGS data were publicly available, using Primer-BLAST. For the assembly, all raw reads were first trimmed using Trimmomatic (Bolger et al., 2014), i.e. ‘Illuminaclip’ set to value ‘NexteraPE-PE.fa:2:30:10’, ‘leading:10’ and ‘trailing:10’, ‘slidingwindow:4:20’, ‘minlen:40’. Trimmed reads were *de novo* assembled using SPAdes (Bankevich et al., 2012) setting the ‘--careful’ and ‘--cov-cutoff 10’ options. Contigs below 1000 bp in length were removed using Seqtk seq^[[1]](#footnote-1)^ using the ‘-L’ option. The obtained *se* gene profile per isolate based on the *in silico* PCR is depicted in Supplementary table S4.

Except for 14 (*selx* in TIAC1991, TIAC1993, TIAC1994, TIAC3152, SAMN02391177, SAMN02403200, and SAMN13134218; *sec*, *seg* and *selx* in TIAC1992; *sem* and *seo* in TIAC3462; and *seq* and *selx* in TIAC3972) of the in total 432 (i.e. 27 genes for each of the 16 isolates) observations, all primer pairs rendered identical results concerning the presence/absence of *se* genes in each isolate (Supplementary Table S4). These 14 observations were related to 16 primer pairs (see Supplementary Table S4: sec-3/5/10/12, seg-2/7/8/9/10, sem-1/3, seo1/2, seq-6, and selx-1/3). Manual alignment of the 16 primer pairs with the assemblies indicated that the inconsistent detection of these 14 genes resulted from: (i) An assembly fragmentation (in 2 observations, i.e. four and five of the ten used primer pairs for *sec* and *seg* in TIAC1992, respectively, annealed on segregated contigs); or (ii) A higher nucleotide variation than accepted by the predefined criteria (in 12 observations, i.e. for two of the three and five used primer pairs detecting *sem* and *seo* in TIAC3462, respectively; for one of the six and three used primer pairs detecting *seq* in TIAC3972, and *selx* in TIAC1991, TIAC1992, TIAC1993, TIAC1994, SAMN02391177, and SAMN13134218, respectively; and for two of the three used primer pairs detecting *selx* in TIAC3152, TIAC3972, and SAMN02403200). In other words, besides the 9 primer pairs that did not lead to an *in silico* product because of assembly fragmentation (i.e. sec-3/5/10/12 and seg-2/7/8/9/10), seven of the 16 primer pairs (5.04% of the 139 used primer pairs, i.e. sem-1/3, seo-1/2, seq-6, and selx-1/3) were determined to specifically detect their target gene, but did not consistently do so when present across the 16 isolates, due to their annealing to non-conservative gene regions (see Supplementary Table S1 marked in yellow). However, since other gene-specific primer pairs were able to detect these 14 genes, they were considered present within the respective isolates (see Materials and Methods in the main manuscript).

Since it was indicated previously that PCR is unable to distinguish between *selu*, *selu2* and pseudogenes *Ψent1* and *Ψent2* (Heymans et al., 2010; Liang et al., 2016) due to their high sequence similarity, the detected presence of the *selu*/*selu2* gene (i.e. in the TIAC1798, TIAC1847, TIAC1848, TIAC1992, TIAC3152, TIAC3462, SAMN02391177, SAMN02403200, and SAMN13134218 isolates) was further investigated through repeating the *in silico* PCR using another primer set (see 1.1 Investigation of specificity of conventional PCR primer pairs) that targets the complete gene length of *selu*, *selu2*, and the pseudogenes. The amplicon sequences were then extracted and aligned against those of NCBI sequences with the aim to type the specific gene (Supplementary Fig. S1). Based on similarity with the reference sequences, it was determined that TIAC1798, TIAC1847, and TIAC1848 contained the *Ψent1-2* genes, TIAC1992, TIAC3152, SAMN02391177 and SAMN02403200 the *selu2* gene, and TIAC3462 and SAMN13134218 the *selu* gene.

# Supplementary Figures and Tables

## Supplementary Figures

| 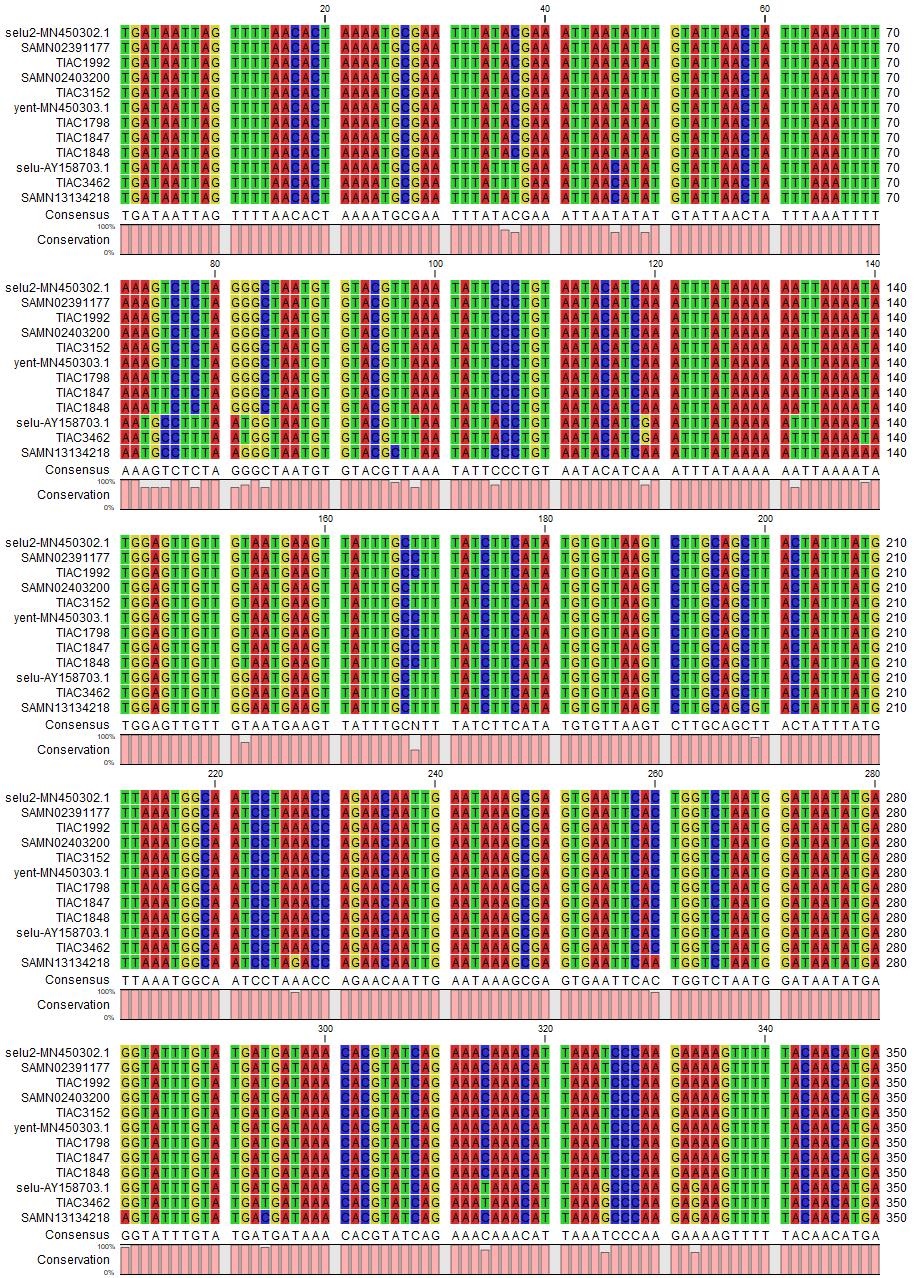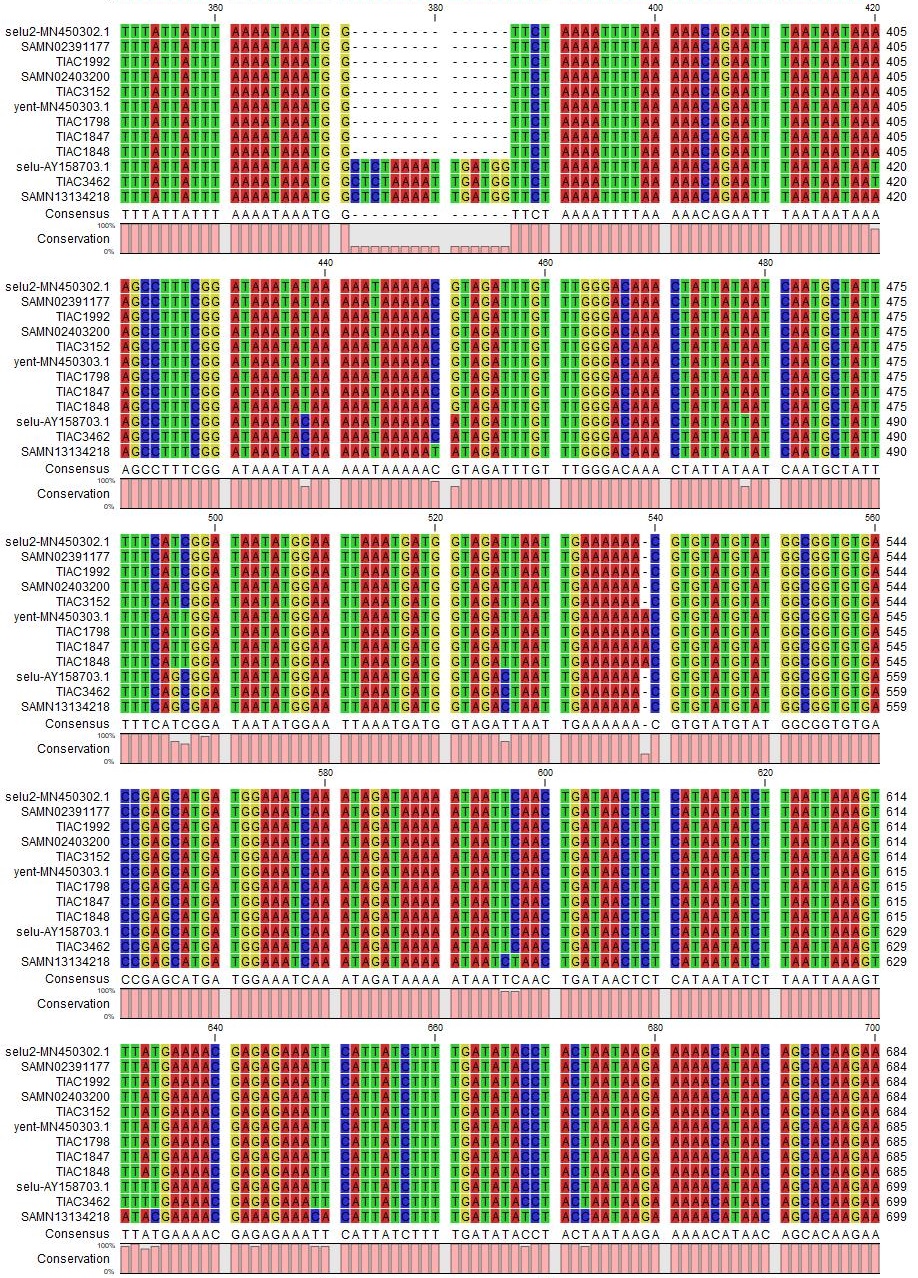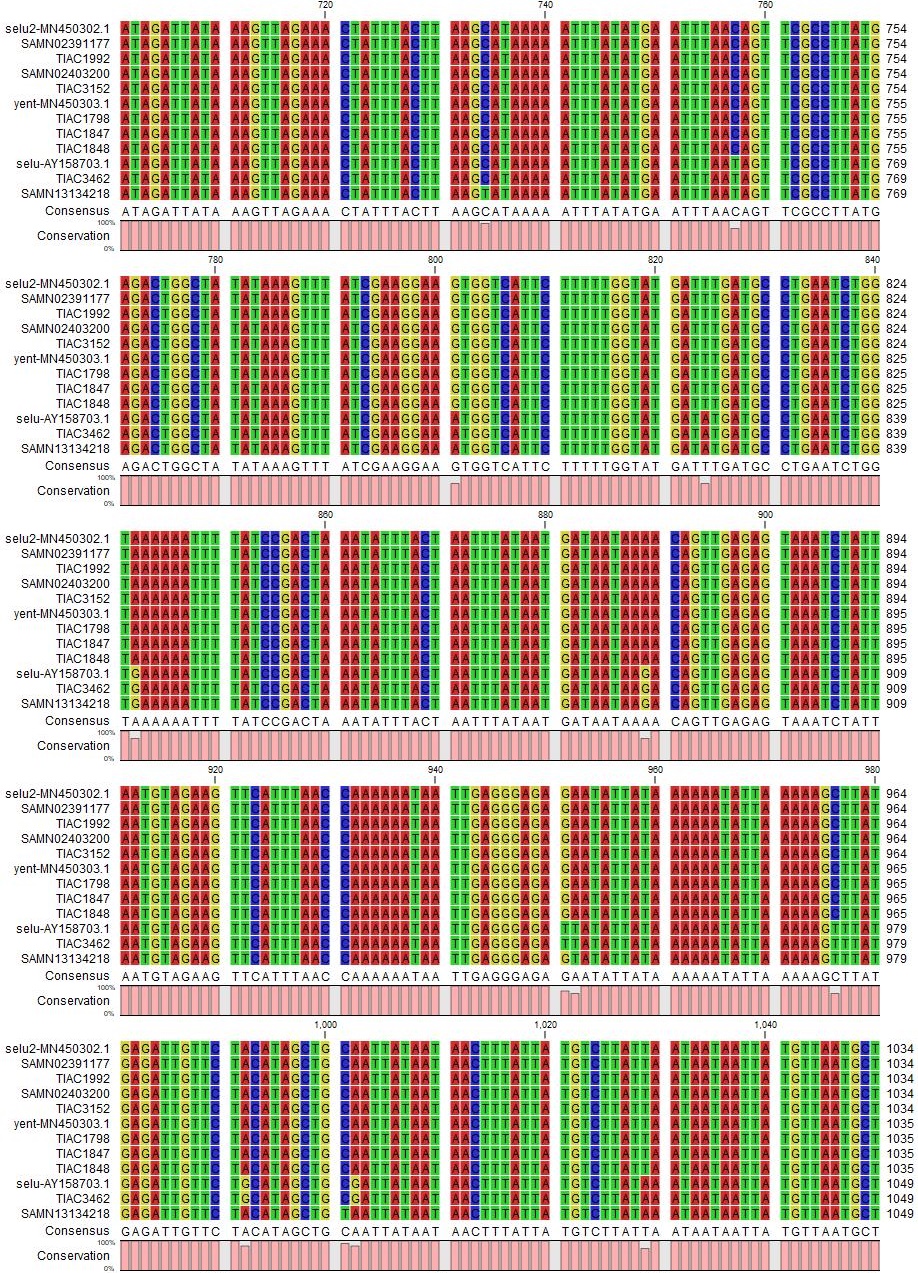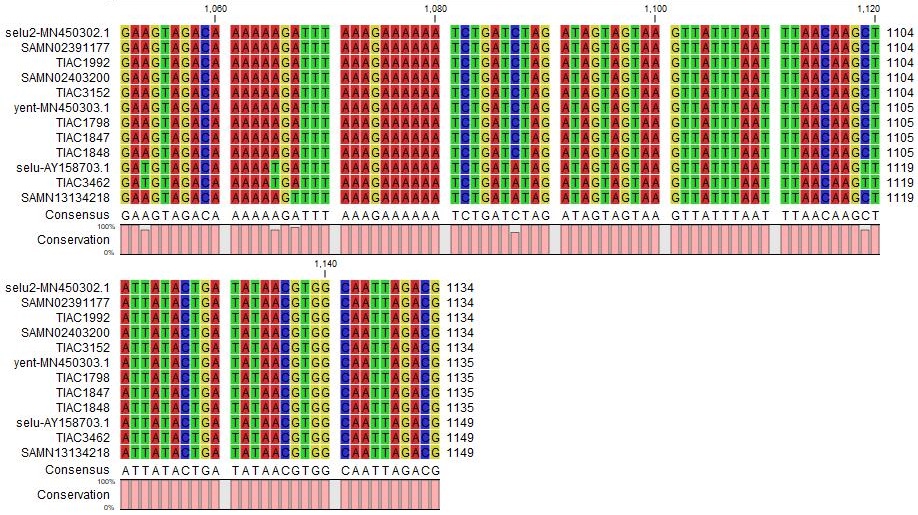 |
| --- |

**Supplementary Figure 1.** Since *Ψent*, *selu*, and *selu2* genes are indistinguishable with PCR, a PCR primer pair covering the complete gene region (forward: 5’- TGA TAA TTA GTT TTA ACA CTA AAA TGC G-3’; reverse: 5’- CGT CTA ATT GCC ACG TTA TAT CAG T-3’; (Letertre et al., 2003)) was used *in silico* in isolates TIAC1798, TIAC1847, TIAC1848, TIAC1992, TIAC3152, TIAC3462, SAMN02391177, SAMN02403200, and SAMN13134218 for which the presence of these genes was indicative (i.e. detected with the *in silico* PCR primer pairs). The amplicons were extracted and aligned against the amplicons of reference sequences for *selu* (GenBank reference AY158703.1), *selu2* (GenBank reference MN450302.1), and *Ψent* (GenBank reference MN450303.1), using CLC Sequence Viewer 8.0. Based on identity of the sequences with the reference sequences, TIAC1798, TIAC1847, and TIAC1848 were identified to contain *Ψent1* and *Ψent2* genes, TIAC1992, TIAC3152, SAMN02403200 and SAMN02391177 to contain the *selu2* gene, and TIAC3462 and SAMN13134218 to contain the *selu* gene.

## Supplementary Tables

Table S1. All conventional PCR primer pairs available in literature for the detection of *se* genes

| **Gene** | **Primer set** | **Forward primer sequence 5'-3'** | **Reverse primer sequence 5'-3'** | **Theoretic amplicon size (bp)** | **Remark** | **Literature** |
| --- | --- | --- | --- | --- | --- | --- |
| *sea* | sea-1 | CCT TTG GAA ACG GTT AAA ACG | TCT GAA CCT TCC CAT CAA AAA C | 127 |  | (Becker et al., 1998) |
|  | sea-2 | TGT ATG TAT GGA GGT GTA AC | ATT AAC CGA AGG TTC TGT | 270 |  | (Sharma et al., 2000) |
|  | sea-3 | CAG CAT ACT ATA TTG TTT AAA GGC | CCT CTG AAC CTT CCC ATC | 400 |  | (Park et al., 2011) |
|  | sea-4 | TTG GAA ACG GTT AAA ACG AA | GAA CCT TCC CAT CAA AAA CA | 120 |  | (Johnson et al., 1991) |
|  | sea-5 | GAA AAA AGT CTG AAT TGC AGG GAA CA | CAA ATA AAT CGT AAT TAA CCG AAG GTT C | 560 |  | (Jarraud et al., 2002) |
|  | sea-6 | CTG TTC AGG AGT TGG ATC TTC | CTT GAG CAC CAA ATA AAT CG | 156 | Aspecific detection of highly similar *sep* and *see* genes | (Nagaraj et al., 2014) |
|  | sea-7 | GCA GGG AAC AGC TTT AGG C | GTT CTG TAG AAG TAT GAA ACA CG | 520 |  | (Monday and Bohach, 1999) |
|  | sea-8 | GGT TAT CAA TGT GCG GGT GG | CGG CAC TTT TTT CTC TTC GG | 102 | Aspecific detection of highly similar *see* gene | (Couch and Betley, 1989) |
|  | sea-9 | GGT TAT CAA TGT GCG GGT GG | CGG CAC TTT TTT CTC TTC GG | 102 | Aspecific detection of highly similar *see* gene; Used by EURL-CPS | (Mehrotra et al., 2000; Roussel et al., 2015) |
|  | sea-10 | CAG CAT ACT ATA TTG TTT AAA G | TCT TAA TAG TGT ATT TGA ATT CT | 489 |  | (Roetzer et al., 2016) |
|  | sea-11 | TAA GGA GGT GGT GCC TAT GG | CAT CGA AAC CAG CCA AAG TT | 180 | Aspecific detection of putative Staphylococcal holin-like toxin | (Cremonesi et al., 2005) |
|  | sea-12 | AAA GTC CCG ATC AAT TTA TGG CT | GTA ATT AAC CGA AGG TTC TGT AGA | 219 |  | (Tsen and Chen, 1992) |
| *seb* | seb-1 | CCA GAT CCT AAA CCA GAT GAG TT | GTT TTT CGT TTG TCA GTT TGA TG | 326 |  | (Shylaja et al., 2010) |
|  | seb-2 | TCG CAT CAA ACT GAC AAA CG | GCA GGT ACT CTA TAA GTG CCT GC | 477 |  | (Becker et al., 1998) |
|  | seb-3 | TGT ATG TAT GGA GGT GTA AC | ATA GTG ACG AGT TAG GTA | 165 |  | (Sharma et al., 2000) |
|  | seb-4 | GTA TGG TGG TGT AAC TGA GCA | TCA ATC TTC ACA TCT TTA GAA TCA | 351 |  | (Park et al., 2011) |
|  | seb-5 | GTA TGG TGG TGT AAC TGA GC | CCA AAT AGT GAC GAG TTA GG | 164 | Used by EURL-CPS | (Mehrotra et al., 2000; Roussel et al., 2015) |
|  | seb-6 | TGA TGA TAA TCA TGT ATC AGC A | ACG GCG ACA CAG TAA CTA TCC A | 851 |  | (Johler et al., 2016) |
|  | seb-7 | TCG CAT CAA ACT GAC AAA CG | GCA GGT ACT CTA TAA GTG CC | 478 |  | (Johnson et al., 1991) |
|  | seb-8 | GTA TAA GAG ATT ATT TAT TTC ACA TG | TAT ATT AAG TCA AAG TAT AGA AAT TG | 231 |  | (Schmitz et al., 1998) |
|  | seb-9 | ATT CTA TTA AGG ACA CTA AGT TAG GGA | ATC CCG TTT CAT AAG GCG AGT | 404 |  | (Jarraud et al., 2002) |
|  | seb-10 | TGC ACA AAT CGA GTA AAT TC | TCA CTT TTT CTT TGT CGT AA | 689 |  | (Roetzer et al., 2016) |
| *sec* | sec-1 | CTC AAG AAC TAG ACA TAA AAG CTA GG | TCA AAA TCG GAT TAA CAT TAT CC | 271 |  | (Becker et al., 1998) |
|  | sec-2 | TGT ATG TAT GGA GGT GTA AC | AAG TAC ATT TTG TAA GTT CC | 69 |  | (Sharma et al., 2000) |
|  | sec-3 | TCA AGA TGC TTA GAA ATC CTC TGT | TCG GTG CTT GCC TTT TTA GGA | 1115 |  | (Johler et al., 2016) |
|  | sec-4 | GAC ATA AAA GCT AGG AAT TT | AAA TCG GAT TAA CAT TAT CC | 257 |  | (Johnson et al., 1991) |
|  | sec-5 | AGA TGA AGT AGT TGA TGT GTA TGG | CAC ACT TTT AGA ATC AAC CG | 451 |  | (Bohach and Schlievert, 1987) |
|  | sec-6 | CTT GTA TGT ATG GAG GAA TAA CAA AAC ATG | CAT ATC ATA CCA AAA AGT ATT GCC GT | 275 |  | (Jarraud et al., 2002) |
|  | sec-7 | CTT GTA TGT ATG GAG GAA TAA CAA | TGC AGG CAT CAT ATC ATA CCA | 283 | Aspecific detection of *selz* | (Monday and Bohach, 1999) |
|  | sec-8 | CCA TCG ATT AAG AAA AGT GTA ACA GCT C | AGC TAG TTC CTT ATA ACA GC | 930 | No detection of *sec* according to preset criteria | (Fitzgerald et al., 2001) |
|  | sec-9 | GTA AAG TTA CAG GTG GCA AAA CTT G | CAT ATC ATA CCA AAA AGT ATT GCC GT | 297 |  | (Jarraud et al., 2002) |
|  | sec-10 | AGA TGA AGT AGT TGA TGT GTA TGG | CAC ACT TTT AGA ATC AAC CG | 451 | Used by EURL-CPS | (Mehrotra et al., 2000; Roussel et al., 2015) |
|  | sec-11 | CCA CTT TGA TAA TGG GAA CTT AC | GAT TGG TCA AAC TTA TCG CCT GG | 270 |  | (Schmitz et al., 1998) |
|  | sec-12 | ACC AGA CCC TAT GCC AGA TG | TCC CAT TAT CAA AGT GGT TTC C | 371 |  | (Cremonesi et al., 2005) |
| *sed* | sed-1 | CTA GTT TGG TAA TAT CTC CTT TAA ACG | TTA ATG CTA TAT CTT ATA GGG TAA ACA TC | 319 |  | (Becker et al., 1998) |
|  | sed-2 | TGT ATG TAT GGA GGT GTA AC | TTC GGG AAA ATC ACC CTT AA | 306 |  | (Sharma et al., 2000) |
|  | sed-3 | TTC GAA ATG CTG ATG GTT GT | AGC TAT CAT CAA TTT CTT TTC AAG C | 1156 |  | (Johler et al., 2016) |
|  | sed-4 | CTA GTT TGG TAA TAT CTC CT | TAA TGC TAT ATC TTA TAG GG | 317 |  | (Johnson et al., 1991) |
|  | sed-5 | CCA ATA ATA GGA GAA AAT AAA AG | ATT GGT ATT TTT TTT CGT TC | 278 | Used by EURL-CPS | (Mehrotra et al., 2000; Roussel et al., 2015) |
|  | sed-6 | GAA TTA AGT AGT ACC GCG CTA AAT AAT ATG | GCT GTA TTT TTC CTC CGA GAG T | 492 |  | (Jarraud et al., 2002) |
|  | sed-7 | GTG GTG AAA TAG ATA GGA CTG C | ATA TGA AGG TGC TCT GTG G | 384 |  | (Monday and Bohach, 1999) |
|  | sed-8 | GAG GTG TCA CTC CAC ACG AA | TGA AGG TGC TCT GTG GAT AAT G | 349 |  | (Varshney et al., 2009) |
|  | sed-9 | TCT GAA TTA AGT AGT ACC GC | ATT CGT AAT TGT TTT TCG GG | 590 |  | (Roetzer et al., 2016) |
|  | sed-10 | TCA ATT CAA AAG AAA TGG CTC A | TTT TTC CGC GCT GTA TTT TT | 339 |  | (Cremonesi et al., 2005) |
|  | sed-11 | GCA GAT AAA AAT CCA ATA ATA GG | ATC TAA AGA AAC TTC TTT TTG TA | 333 |  | (Tsen and Chen, 1992) |
| *see* | see-1 | CAG TAC CTA TAG ATA AAG TTA AAA CAA GC | TAA CTT ACC GTG GAC CCT TC | 178 |  | (Becker et al., 1998) |
|  | see-2 | TGT ATG TAT GGA GGT GTA AC | GCC AAA GCT GTC TGA G | 213 | Used by EURL-CPS | (Sharma et al., 2000; Roussel et al., 2015) |
|  | see-3 | TAG ATA AAG TTA AAA CAA GC | TAA CTT ACC GTG GAC CCT TC | 170 |  | (Johnson et al., 1991) |
|  | see-4 | CAA AGA AAT GCT TTA AGC AAT CTT AGG C | CAC CTT ACC GCC AAA GCT G | 482 |  | (Jarraud et al., 2002) |
|  | see-5 | TAC CAA TTA ACT TGT GGA TAG AC | CTC TTT GCA CCT TAC CGC | 170 |  | (Monday and Bohach, 1999) |
|  | see-6 | AGG TTT TTT CAC AGG TCA TCC | CTT TTT TTT CTT CGG TCA ATC | 209 | Aspecific detection of highly similar *sep* gene | (Couch et al., 1988) |
|  | see-7 | ACC GAT TGA CCG AAG AAA AA | ATT GCC CTT GAG CAT CAA AC | 264 | Aspecific detection of highly similar *sea* gene | (Varshney et al., 2009) |
|  | see-8 | CAA AGA AAT GCT TTA AGC AAT CTT AGG CCA C | CTT ACC GCC AAA GCT G | 482 | No detection of *see* according to preset criteria | (Jarraud et al., 1999) |
|  | see-9 | TTA CAA AGA AAT GCT TTA AGC | TAA ACC AAA TTT TCC GT | 456 |  | (Tsen and Chen, 1992) |
| *seg* | seg-1 | AAG TAG ACA TTT TTG GCG TTC C | AGA ACC ATC AAA CTC GTA TAG C | 287 |  | (Omoe et al., 2002) |
|  | seg-2 | GGT TCA TTG TCA AAT AGA CTG | CTA TTG TCG ATT GTT ACC TG | 520 |  | (Nagaraj et al., 2014) |
|  | seg-3 | GTT AGA GGA GGT TTT ATG | TTC CTT CAA CAG GTG GAG A | 198 | Used by EURL-CPS | (Bania et al., 2006; Roussel et al., 2015) |
|  | seg-4 | TCT CCA CCT GTT GAA GG | AAG TGA TTG TCT ATT GTC G | 323 |  | (Holtfreter et al., 2007) |
|  | seg-5 | TGC TAT CGA CAC ACT ACA ACC | CCA GAT TCA AAT GCA GAA CC | 704 |  | (Mclauchlin et al., 2000) |
|  | seg-6 | CGTCTCCACCTGTTGAAGG | CCA AGT GAT TGT CTA TTG TCG | 327 |  | (Monday and Bohach, 1999) |
|  | seg-7 | TAA GGG AAC TAT GGG TAA TGT AAT G | GAA CAA AAG GTA CTA GTT CTT TTT TAG G | 561 |  | (Thomas et al., 2006) |
|  | seg-8 | AAT TAT GTG AAT GCT CAA CCC GAT C | AAA CTT ATA TGG AAC AAA AGG TAC TAG TTC | 642 |  | (Jarraud et al., 1999) |
|  | seg-9 | GTT GAA GGA AGA GGA GTT AT | TCA ACA ACT TTA TTA TCT CCG | 558 |  | (Roetzer et al., 2016) |
|  | seg-10 | CCA CCT GTT GAA GGA AGA GG | TGC AGA ACC ATC AAA CTC GT | 432 |  | (Cremonesi et al., 2005) |
| *seh* | seh-1 | CAA CTG CTG ATT TAG CTC AG | CCC AAA CAT TAG CAC CA | 173 | Used by EURL-CPS | (Bania et al., 2006; Roussel et al., 2015) |
|  | seh-2 | CAA CTG CTG ATT TAG CTC AG | GTC GAA TGA GTA ATC TCT AGG | 360 |  | (Monday and Bohach, 1999) |
|  | seh-3 | GTC TAT ATG GAG GTA CAA CAC T | GAC CTT TAC TTA TTT CGC TGT C | 213 |  | (Omoe et al., 2002) |
|  | seh-4 | CAA TCA CAT CAT ATG CGA AAG CAG | CAT CTA CCC AAA CAT TAG CAC C | 376 |  | (Jarraud et al., 2002) |
|  | seh-5 | CGA AAG CAG AAG ATT TAC ACG | GAC CTT TAC TTA TTT CGC TGT C | 495 |  | (Mclauchlin et al., 2000) |
|  | seh-6 | TAG CTA ATG CAT ATG GTC AA | AGA TTT TAA AGT TTT ATT GTC TTC A | 563 |  | (Roetzer et al., 2016) |
|  | seh-7 | TCA CAT CAT ATG CGA AAG CAG | TCG GAC AAT ATT TTT CTG ATC TTT | 463 |  | (Cremonesi et al., 2005) |
| *sei* | sei-1 | GGC CAC TTT ATC AGG ACA | AAC TTA CAG GCA GTC CA | 328 | Used by EURL-CPS | (Bania et al., 2006; Roussel et al., 2015) |
|  | sei-2 | GGT GAT ATT GGT GTA GGT AAC | ATC CAT ATT CTT TGC CTT TAC CAG | 454 |  | (Omoe et al., 2002) |
|  | sei-3 | GGT GAT ATT GGT GTA GGT AA | CAT ATT CTT TGC CTT TAC CAG | 451 |  | (Nagaraj et al., 2014) |
|  | sei-4 | CTY GAA TTT TCA ACM GGT AC | AGG CAG TCC ATC TCC TG | 461 |  | (Holtfreter et al., 2007) |
|  | sei-5 | GAC AAC AAA ACT GTC GAA ACT G | CCA TAT TCT TTG CCT TTA CCA G | 630 |  | (Mclauchlin et al., 2000) |
|  | sei-6 | CAA CTC GAA TTT TCA ACA GGT AC | CAG GCA GTC CAT CTC CTG | 465 |  | (Monday and Bohach, 1999) |
|  | sei-7 | TGG AAC AGG ACA AGC TGA AA | TGT TTG CCA TTA ACC CAA AG | 529 |  | (Varshney et al., 2009) |
|  | sei-8 | CTC AAG GTG ATA TTG GTG TAG G | GTT ACT ATC TAC ATA TGA TAT TTCGAC ATC | 656 |  | (Thomas et al., 2006) |
|  | sei-9 | CTC AAG GTG ATA TTG GTG TAG G | AAA AAA CTT ACA GGC AGT CCA TCT C | 576 |  | (Jarraud et al., 2002) |
|  | sei-10 | CTA TTG CAA ATC AAC TCG AA | AAA AAC TTA CAG GCA GTC C | 486 |  | (Roetzer et al., 2016) |
|  | sei-11 | CTC AAG GTG ATA TTG GTG TAG G | CAG GCA GTC CAT CTC CTG TA | 529 |  | (Cremonesi et al., 2005) |
| *selj* | selj-1 | ATA GCA TCA GAA CTG TTG TTC CG | CTT TCT GAA TTT TAC CAC CAA AGG | 152 |  | (Omoe et al., 2005) |
|  | selj-2 | GTT CTG GTG GTA AAC CA | GCG GAA CAA CAG TTC TGA | 131 | Used by EURL-CPS | (Bania et al., 2006; Roussel et al., 2015) |
|  | selj-3 | TCA GAA CTG TTG TTC CGC TAG | GAA TTT TAC CAY CAA AGG TAC | 138 |  | (Holtfreter et al., 2007) |
|  | selj-4 | CAT CAG AAC TGT TGT TCC GCT AG | CTG AAT TTT ACC ATC AAA GGT AC | 142 |  | (Monday and Bohach, 1999) |
|  | selj-5 | TAA CCT CAG ACA TAT ATA CTT CTT TAA CG | AGT ATC ATA AAG TTG ATT GTT TTC ATG CAG | 300 | Divergent amplicon size: 295bp | (Jarraud et al., 2002) |
|  | selj-6 | ACG AAA AGG GTA TCT CTG AA | ACA GAA CCA AAG GTA GAC TT | 636 |  | (Roetzer et al., 2016) |
|  | selj-7 | GGT TTT CAA TGT TCT GGT GGT | AAC CAA CGG TTC TTT TGA GG | 306 |  | (Cremonesi et al., 2005) |
| *sek* | sek-1 | TAG GTG TCT CTA ATA ATG CCA | TAG ATA TTC GTT AGT AGC TG | 293 |  | (Omoe et al., 2005) |
|  | sek-2 | GGA GAA AAG GCA ATG AA | TAG TGC CGT TAT GTC CA | 516 |  | (Bania et al., 2006) |
|  | sek-3 | CGA CAT CCA AAT GGA ATT TCT CAG ACT CTA CAG | GCA GAG AAT TTT CAT TTG GAT GTA GAG ATT TCA TAT GAG | 46 | Aspecific detection of *selq* | (Aguilar et al., 2014) |
|  | sek-4 | ATG CCA GCG CTC AAG GC | AGA TTC ATT TGA AAA TTG TAG TTG ATT AGC T | 134 |  | (Holtfreter et al., 2007) |
|  | sek-5 | AGG AAT TGA TAA TCT CAG GA | CCA AAT GGA ATT TCT CAG AC | 617 |  | (Roetzer et al., 2016) |
| *sel* | sel-1 | TAAC GGC GAT GTA GGT CCA GG | CAT CTA TTT CTT GTG CGG TAA C | 383 |  | (Omoe et al., 2005) |
|  | sel-2 | CGA TGT AGG TCC AGG A | TTC TTG TGC GGT AAC CA | 369 |  | (Bania et al., 2006) |
|  | sel-3 | GCG ATG TAG GTC CAG GAA AC | CAT ATA TAG TAC GAG AGT TAG AAC CAT A | 234 |  | (Holtfreter et al., 2007) |
|  | sel-4 | AAA ATT CAC CAG AAT CAC AC | TTA AGA AGC TTT CTG GAA GA | 501 |  | (Roetzer et al., 2016) |
|  | sel-5 | CAC CAG AAT CAC ACC GCT TA | CTG TTT GAT GCT TGC CAT TG | 240 |  | (Cremonesi et al., 2005) |
| *sem* | sem-1 | GGA TAA TTC GAC AGT AAC AG | TCC TGC ATT AAA TCC AGA AC | 379 |  | (Omoe et al., 2005) |
|  | sem-2 | CAT ATC GCA ACC GCT GA | TCA GCT GTT ACT GTC GA | 148 | Aspecific detection of *selv* | (Bania et al., 2006) |
|  | sem-3 | CTA TTA ATC TTT GGG TTA ATG GAG AAC | TTC AGT TTC GAC AGT TTT GTT GTC AT | 300 | Divergent amplicon size: 326bp | (Jarraud et al., 2002) |
|  | sem-4 | GAT AAT TCG ACA GTA ACA GC | CGA CAG TTT TGT TGT CAT TA | 490 |  | (Roetzer et al., 2016) |
| *sen* | sen-1 | TAT GTT AAT GCT GAA GTA GAC | ATT TCC AAA ATA CAG TCC ATA | 282 |  | (Omoe et al., 2005) |
|  | sen-2 | CAT CAT GCT TAT ACG GAG GAG | CCC ACT GAA CCT TTT ACG TT | 301 |  | (Park et al., 2011) |
|  | sen-3 | GGC AAT TAG ACG AGT CA | ATC GTA ACT CCT CCG TA | 237 |  | (Bania et al., 2006) |
|  | sen-4 | CGT GGC AAT TAG ACG AGT C | GAT TGA TYT TGA TGA TTA TKA G | 474 |  | (Holtfreter et al., 2007) |
|  | sen-5 | ACG TGG CAA TTA GAC GAG TC | GAT TGA TCT TGA TGA TTA TGA GAA TGA AAG | 476 |  | (Thomas et al., 2006) |
|  | sen-6 | CTT CTT GTT GGA CAC CAT CTT | GAA ATA AAT GTG TAG GCT T | 135 |  | (Chiang et al., 2008) |
|  | sen-7 | ATG AGA TTG TTC TAC ATA GCT GCA AT | AAC TCT GCT CCC ACT GAA C | 680 |  | (Jarraud et al., 2002) |
|  | sen-8 | TAC TGA TAT AAC GTG GCA AT | AGA TGA GCT AAC TGT TCT ATT | 574 |  | (Roetzer et al., 2016) |
| *seo* | seo-1 | TGT GTA AGA AGT CAA GTG TAG | TCT TTA GAA ATC GCT GAT GA | 214 |  | (Omoe et al., 2005) |
|  | seo-2 | GTC AAG TGT AGA CCC TA | TGT ACA GGC AGT ATC CA | 288 |  | (Bania et al., 2006) |
|  | seo-3 | AGT TTG TGT AAG AAG TCA AGT GTA GA | ATC TTT AAA TTC AGC AGA TAT TCC ATC TAA C | 180 |  | (Jarraud et al., 2002) |
|  | seo-4 | AAA TTC AGC AGA TAT TCC AT | TTT GTG TAA GAA GTC AAG TGT AG | 172 |  | (Chiang et al., 2008) |
|  | seo-5 | TTG TGT AAG AAG TCA AGT GT | GAT AGT CTG ATG AAT CTA TTG TT | 646 |  | (Roetzer et al., 2016) |
| *sep* | sep-1 | TGA TTT ATT AGT AGA CCT TGG | ATA ACC AAC CGA ATC ACC AG | 396 |  | (Omoe et al., 2005) |
|  | sep-2 | TCA AAA GAC ACC GCC AA | ATT GTC CTT GAG CAC CA | 396 | Used by EURL-CPS | (Bania et al., 2006; Roussel et al., 2015) |
|  | sep-3 | GAA TTG CAG GGA ACT GCT | GGC GGT GTC TTT TGA AC | 182 |  | (Holtfreter et al., 2007) |
|  | sep-4 | ATC ATA ACC AAC CGA ATC AC | AGA AGT AAC TGT TCA GGA GCT A | 148 |  | (Chiang et al., 2008) |
|  | sep-5 | GAC CTT GGT TCA AAA GAC ACC | TGT CTT GAC TGA AGG TCT AGC | 275 |  | (Roetzer et al., 2016) |
| *seq* | seq-1 | AAT CTC TGG GTC AAT GGT AAG C | TTG TAT TCG TTT TGT AGG TAT TTT CG | 122 |  | (Omoe et al., 2005) |
|  | seq-2 | TCA AGG AGT TAG TTC TGG AAA TT | GCT TAC CAT TGA CCC AGA GA | 251 |  | (Park et al., 2011) |
|  | seq-3 | GGA ATT ACG TTG GCG AA | AAC TCT CTG CTT GAC CA | 330 |  | (Bania et al., 2006) |
|  | seq-4 | ACC TGA AAA GCT TCA AGG A | CGC CAA CGT AAT TCC AC | 204 |  | (Holtfreter et al., 2007) |
|  | seq-5 | TCA GGT CTT TGT AAT ACA AAA | TCT GCT TGA CCA GTT CCG GT | 359 |  | (Chiang et al., 2008) |
|  | seq-6 | AAA AGC TTC AAG GAG TTA GT | ATC CAA ATG AAA ATT CTC TGC | 572 |  | (Roetzer et al., 2016) |
| *ser* | ser-1 | GGA TAA AGC GGT AAT AGC AG | GTA TTC CAA ACA CAT CTA AC | 166 |  | (Omoe et al., 2005) |
|  | ser-2 | AGA TGT GTT TGG AAT ACC CTA T | CTA TCA GCT GTG GAG TGC AT | 123 | Used by EURL-CPS | (Chiang et al., 2008; Roussel et al., 2015) |
|  | ser-3 | AGC GGT AAT AGC AGA AAA TG | TCT TGT ACC GTA ACC GTT TT | 363 |  | (Holtfreter et al., 2007; Varshney et al., 2009) |
| *ses* | ses-1 | TGA ATT AGA TTC AAC CGC AC | CGT CTA TGT GTA AAT TTG AAG AG | 623 |  | (Roetzer et al., 2016) |
|  | ses-2 | TTC AGA AAT AGC CAA TCA TTT CAA | CCT TTT TGT TGA GAG CCG TC | 195 |  | (Ono et al., 2008) |
| *set* | set-1 | CGA ATC AAT ACA TTA GAC GA | TTG TGT AAT CAA GTG TAA AGT | 418 |  | (Roetzer et al., 2016) |
|  | set-2 | GGT GAT TAT GTA GAT GCT TGG G | TCG GGT GTT ACT TCT GTT TGC | 170 |  | (Ono et al., 2008) |
| *selu* | selu-1 | ATC AGA AAC AAA CAT TAA AGC CCA | TGA CCA TTT CCT TCG ATA AAC TTT AT | *selu*: 500 *Ψent1-2*/*selu2*: 486 | Aspecific detection of *Ψent1-2* pseudogenes | (Park et al., 2011) |
|  | selu-2 | AAT GGC TCT AAA ATT GAT GG | ATT TGA TTT CCA TCA TGC TC | 215 | Detection *selu* only (no *selu2*) | (Holtfreter et al., 2007; Varshney et al., 2009) |
|  | selu-3 | ATT TGC TTT TAT CTT CAT | GGA CTT TAA TGT TTG TTT CTG AT | 167 | Aspecific detection of *Ψent1-2* pseudogenes | (Chiang et al., 2008) |
|  | selu-4 | TAA AAT AAA TGG CTC TAA AAT TGA TGG | CGT CTA ATT GCC ACG TTA TAT CAG T | 790 | Detection *selu* only (no *selu2*) | (Letertre et al., 2003) |
|  | selu-5 | TGA TAA TTA GTT TTA ACA CTA AAA TGC G | CGT CTA ATT GCC ACG TTA TAT CAG T | *Ψent1-2*/*selu2:* 1135 *selu:* 1149 | Aspecific detection of *Ψent1-2* pseudogenes | (Letertre et al., 2003) |
|  | selu-6 | TAA AAT AAA TGG CTC TAA AAT TGA TGG | ATC CGC TGA AAA ATA GCA TTG AT | 142 | Detection *selu* only (no *selu2*) | (Letertre et al., 2003) |
| *selv* | selv-1 | GCAGGATCC GAT GTC GGA GTT TTG AAT CTT AGG | TAACTGCAG TTA GTT ACT ATC TAC ATA TGA TAT TTC GAC ATC | *selv:* 653  *sem*-*i:* 1417 | Cloning primers; Detection of *sem* and *sei,* or *selv* at different amplicon sizes | (Thomas et al., 2006) |
| *selw* | selw-1 | GGA ATT TTT ACA AAT TCA GCG | CTA CAT TGC GTT TTA TTG GTT G | 323 |  | (Aung et al., 2017) |
|  | selw-2 | TTG TTT TGG GGG AGT TTG AAG | GTC AAA GAT TAT TAA TGA TTA AC | ca. 800 |  | (Aung et al., 2019) |
|  | selw-3 | TTG TTT TGG GGG AGT TTG AAG | ACT TTA TAT TTC ACT GTC AAA G | ca. 800 |  | (Aung et al., 2019) |
|  | selw-4 | ATT TTT ACA AAT TCA GCG AGT | TAA CAC CAC CAT AAC TAC AT | 334 |  | (Roetzer et al., 2016) |
| *selx* | selx-1 | GTC CAA TTA TGT GTA GAC GA | GAA TTT TCT ATA TGA TGG TGC T | ca. 900 |  | (Aung et al., 2019) |
|  | selx-2 | AGC AGA CGC GTC AAC ACA AA | ACT TGT TCA ATG TCA TTA ACA CTT TTC AC | 513 |  | (Wilson et al., 2011) |
|  | selx-3 | TTA CGA TAG ATA CAG CAA GG | TGT CAT TAA CAC TTT TCA CAA | 401 |  | (Roetzer et al., 2016) |
| *sey* | sey-1 | CAA TGT ACG GAC AGT GCT CTA CAA | TGA CCG TTA ACA AAC AAG TTC ATT C | 189 |  | (Ono et al., 2015) |
|  | sey-2 | GAA ATA TTG ATA TAT AGA TCA TT | CCT AAG AAC TTA ATK TCC TAA GC | 843 | Aspecific detection of *mvaS* | (Aung et al., 2019) |
| *selz* | selz-1 | GGT TAC AGT AGC TAT TCT TTG TTG | GTA AAC TTT ACA ACA ATA GGC TG | 443 |  | (Aung et al., 2019) |
|  | selz-2 | GTT ATA AAT AGC AAT GGT TG | GAT AAG TTG CTC TAT GTC TA | ca. 800 | No detection of *selz* according to preset criteria | (Aung et al., 2019) |
|  | selz-3 | GTT ATA AAT AGC AAT GGT TG | GAG CAA CTT TTC CAA GTC GC | ca. 1000 | No detection of *selz* according to preset criteria | (Aung et al., 2019) |
| *sel26* | sel26-1 | CTT ATG CTG ATG TAG GTG TTC | CAT TTA TCC AAA GAT TTA TCG G | 312 |  | (Aung et al., 2019) |
| *sel27* | sel27-1 | TGA AGG CGC TCT ATG AAT CAG | TAC TCT GCG TAA AAT TTG GG | 500 |  | (Aung et al., 2019) |

A literature study was performed to collect all published conventional PCR primer sets for the detection of *se* genes. For each identified primer set, the sequences of the primers, the theoretic amplicon size and the reference to the respective study is shown. When the theoretic amplicon size was not known or indicated in the respective study, the amplicon size was manually determined by aligning the primers against NCBI sequences using BLAST, and is indicated in blue. When differences in the amplicon length were observed with those published in the respective study, it is indicated in the column ‘Remark’. All primer pairs were evaluated for their specificity to the target gene with BLAST against the NCBI nucleotide collection for *S. aureus* (taxid: 1280). Primer pairs shown in grey were not used for the *in silico* PCR because they rendered aspecific results, i.e. false positive detection of other Staphylococcal genes or no detection of the target gene in any of the NCBI sequences within the *S. aureus* collection, based on the predefined criteria. Their aspecificity is in more detail explained in the column ‘Remark’. Because conventional PCR detection does not enable the discrimination between *selu*, *selu2* and pseudogenes *Ψent1* and *Ψent2* due to high sequence similarity, one of the *selu* primer pairs was randomly selected for the *in silico* PCR (shown in light green). Primer pairs shown in yellow (i.e. 5.04% of the 139 primer pairs used for the *in silico* PCR) were used for the *in silico* PCR to determine the *se* gene profile in the 13 isolates and three publicly available WGS data sets, but were observed to not always detect the target gene when present because of an increased number of mismatches compared to those accepted by the preset criteria (e.g. due to *se* variants), in contrast to the other gene-specific primer pairs (see Supplementary Data). Since no primer pairs for PCR were described in literature, cloning primers described in literature were used for *selv* (by removing the 5’ end restriction sites underlined in the corresponding sequences). It is also mentioned in the column ‘Remark’ which primer sets are applied by the EURL-CPS.

Table S2. Overview of selected public data

| **NCBI BioSample accession** | **Genbank assembly accession** | **Raw reads available (SRA experiment ID)** | ***se* gene profile** | **Reference** |
| --- | --- | --- | --- | --- |
| SAMN02391177 | GCA_000636155.1 | [yes (SRX546124)](https://www.ncbi.nlm.nih.gov/sra/SRX546124%5baccn%5d) | *seg, sei, sen, seo, selu, selx, sey, sel26, sel27* | (Merda et al., 2020) |
| SAMN02403200 | GCA_000586795.1 | yes (SRX468300) | *seg, sei, selj, sem, sen, seo, ser, ses, set, selu* | (Merda et al., 2020) |
| [SAMN13134218](https://www.ncbi.nlm.nih.gov/biosample/SAMN13134218/) | GCA_009696775.1 | no | *seg, sei, sem, sen, seo, selu, selv* | (Fursova et al., 2020) |

For the three isolates of which WGS data were publicly available and used in this study to cover all described se genes within our study, the NCBI BioSample accession and GenBank accession of the assembly are shown. When raw reads were also available, the SRA experiment ID is shown. For all public data, the *se* gene profiles as obtained by the respective studies are indicated. The public data were selected because of the previously described presence of *se* genes shown in blue.

Table S3. Assembly statistics

| **Isolate name** | **DNA extraction kit** | **N50** | **Number of contigs** | **Median coverage against assembly** |
| --- | --- | --- | --- | --- |
| TIAC1840 | GenElute-NL | 536,480 | 16 | 57 |
| TIAC1848 | GenElute-NL | 510,559 | 19 | 56 |
| TIAC1991 | GenElute-NL | 977,656 | 11 | 59 |
| TIAC1992 | GenElute-NL | 168,338 | 28 | 67 |
| TIAC1993 | GenElute-NL | 706,096 | 13 | 75 |
| TIAC1994 | GenElute-NL | 505,583 | 13 | 81 |
| TIAC2001 | GenElute-NL | 182,240 | 28 | 75 |
| TIAC3462 | GenElute-NL | 150,369 | 45 | 60 |
| TIAC3971 | GenElute-NL | 416,754 | 19 | 64 |
| TIAC3972 | GenElute-NL | 616,298 | 14 | 66 |
| TIAC1798 | GenElute-NL | 301,818 | 24 | 48 |
|  | GenElute | 195,799 | 27 | 76 |
|  | DNeasy | 208,988 | 25 | 68 |
|  | Wizard | 208,153 | 22 | 60 |
| TIAC1847 | GenElute-NL | 301,817 | 23 | 62 |
|  | GenElute | 197,001 | 40 | 70 |
|  | DNeasy | 241,620 | 23 | 74 |
|  | Wizard | 196,364 | 27 | 57 |
| TIAC3152 | GenElute-NL | 327,865 | 15 | 74 |
|  | GenElute | 267,782 | 20 | 61 |
|  | DNeasy | 477,148 | 14 | 50 |
|  | Wizard | 327,868 | 18 | 81 |

For each sample (i.e. WGS data of an isolate processed with different DNA extraction kits), the N50, number of contigs, and median coverage against the assembly are shown, respectively. The names of the DNA extraction kits are abbreviated, i.e. GenElute-NL: GenElute Bacterial gDNA kit using the protocol for Gram positive bacteria (without lysostaphin); GenElute: GenElute Bacterial gDNA kit using the protocol for Staphylococcal species (with lysostaphin); DNeasy: DNeasy Blood & Tissue kit; Wizard: Wizard gDNA Purification kit.

Table S4. Overview of *se* genes within extended VFDB_Full database

| **Locus** | **Variant** | **Source** | **RefSeq and/or GenBank accession number(s) to corresponding protein sequences** |
| --- | --- | --- | --- |
| *sea** | - | Original VFDB_Full | WP_000750412.1; WP_000750406.1 |
| *seb* | - | Original VFDB_Full | AAA88550.1 |
| *sec*** | *sec1* | NCBI: added to the database | ANJ16441.1 |
|  | *sec2* | NCBI: added to the database | AJP29264.1 |
|  | *sec3* | NCBI: added to the database | BAB43097.3 |
|  | *selc4* | NCBI: added to the database | BAB94624.1 |
|  | *selc_bov_* | NCBI: added to the database | CAI80051.1 |
|  | *selc_ov_* | NCBI: added to the database | ADI96966.1 |
| *sed* | - | Original VFDB_Full | AAB06195.1 |
| *see* | - | Original VFDB_Full | AAA26617.1 |
| *seg** | - | Original VFDB_Full | WP_000736712.1; WP_000736707.1 |
| *seh* | - | Original VFDB_Full | BAB93916.1; WP_000608674.1 |
| *sei* | - | Original VFDB_Full | WP_000713850.1; WP_000713847.1; WP_000721567.1 |
| *selj* | - | Original VFDB_Full | BAC97796.1 |
| *sek* | - | Original VFDB_Full | WP_000733771.1; WP_000734020.1; WP_000733775.1 |
| *sel* | - | Original VFDB_Full | WP_000746597.1; WP_000746599.1; WP_000746599.1 |
| *sem* | - | Original VFDB_Full | WP_000821658.1; WP_000821649.1 |
| *sen** | - | Original VFDB_Full | WP_001236366.1; WP_001235656.1; WP_001236362.1 |
| *seo* | - | Original VFDB_Full | WP_000935742.1; WP_010922839.1; WP_000935739.1; WP_000935747.1 |
| *sep*** | - | NCBI: added to the database | NP_835571.1 |
| *seq* | - | Original VFDB_Full | WP_001033320.1; WP_001033321.1; WP_001033316.1 |
| *ser* | - | Original VFDB_Full | BAC97795.1 |
| *ses* | - | NCBI: added to the database | BAG06667.1 |
| *set* | - | NCBI: added to the database | BAG06666.1 |
| *selu* | *selu* | Original VFDB_Full | WP_000764692.1; WP_000764684.1 |
|  | *selu2* | Original VFDB_Full | ABK27166.1 |
| *selv* | - | Original VFDB_Full | ABK27165.1 |
| *selw* | - | NCBI: added to the database | QKD77131.1; QKO26035.1; QKD77152.1 |
| *selx* | - | NCBI: added to the database | QKD77102.1; QKO25999.1; AEI60189.1; AEI60187.1; AEI60188.1; AEI60185.1; AEI60186.1 |
| *sey* | - | NCBI: added to the database | BAS21358.1 |
| *selz* | - | NCBI: added to the database | QFG75941.1 |
| *sel26* | - | NCBI: added to the database | AVX35650.1; AVX35641.1 |
| *sel27* | - | NCBI: added to the database | AVX35651.1; AVX35642.1 |
| *Ψent1*** | - | NCBI: added to the database | CAC8477980.1 |
| *Ψent2* | - | Original VFDB_Full | WP_001796875.1 |

The VFDB_Full database was extracted from its source at November 16th, 2020. For all *se* genes not present within the database, NCBI sequences were added. Other virulence genes present within this database (related to Staphylococcal and other species) were disregarded and not shown, since they were not further discussed in this study. The source of the sequence (i.e. originally present in the VFDB_Full database, or sequences from NCBI added to the VFDB_Full database) is shown. Since the (Refseq and Genbank) accession numbers of reference sequences in the original VFDB_Full database refer to their corresponding protein sequences, the same nomenclature was extended to all sequences added to the database as well. Moreover, if a RefSeq record in the VFDB_Full database was removed and replaced by another RefSeq accession number on NCBI (identical protein sequence), the most recent accession number was shown. Staphylococcal enterotoxin sequences in the database for which the RefSeq record was suppressed on NCBI because it was no longer annotated on any genome (WP_001797618.1 for *sea*, WP_011382313.1 for *seg*, WP_010922838.1 for *sen*, and WP_000034846.1 for *sep*), were removed in the extended VFDB_Full database (*), and if necessary (i.e. when none of the respective *se* gene remains present in the VFDB_Full database) replaced by a new reference sequence (**). Nucleotide sequences for all *se* reference genes present in the extended VFDB_Full database can be found in the accompanied FASTA file. For this, the nucleotide sequences of all reference genes originally present in the VFDB_Full database were extracted from the original VFDB_Full database and added in the FASTA file, however, corresponding accession numbers to the nucleotide sequences are not available and could thus not be provided. For *se* genes added to the extended VFDB_Full database, the nucleotide sequences and respective nucleotide RefSeq and/or GenBank accession numbers are provided in the FASTA file. The extended VFDB_Full database allows subtyping of *sec* and *selu*. Since variants for *sec* were not all specifically annotated in the database (i.e. the variant was ambiguously described and sequences for some variants were missing), all present *sec* sequences in the VFDB_Full database were removed, and those described in a recently published article were added (Etter et al., 2020).

Table S5. Definitions of True Positives (TP), True Negatives (TN), False Positives (FP) and False Negatives (FN) applied in this study

|  | | ***in silico* PCR** | |
| --- | --- | --- | --- |
|  |  | **Detected** | **Not detected** |
| **WGS** | **Detected** | **True Positive (TP)** Gene detected by WGS (BLAST+ and/or SRST2) and PCR | **False Positive (FP)** Gene detected by WGS (BLAST+ and/or SRST2), but not by PCR |
|  | **Not detected** | **False Negative (FN)** Gene detected by PCR while missed by WGS (BLAST+ and SRST2) | **True Negative (TN)** Gene detected by neither WGS (BLAST+ and SRST2) nor PCR |

Table S6. *se* gene profile determined with *in silico* PCR

| **Isolate** | ***sea*** | ***seb*** | ***sec*** | ***sed*** | ***see*** | ***seg*** | ***seh*** | ***sei*** | ***selj*** | ***sek*** | ***sel*** | ***sem*** | ***sen*** | ***seo*** | ***sep*** | ***seq*** | ***ser*** | ***ses*** | ***set*** | ***selu*** | | ***selv*** | ***selw*** | ***selx*** | ***sey*** | ***Selz*** | ***sel26*** | ***sel27*** | ***Ψent1*** | ***Ψent2*** |
| --- | --- | --- | --- | --- | --- | --- | --- | --- | --- | --- | --- | --- | --- | --- | --- | --- | --- | --- | --- | --- | --- | --- | --- | --- | --- | --- | --- | --- | --- | --- |
|  |  |  |  |  |  |  |  |  |  |  |  |  |  |  |  |  |  |  |  | ***u*** | ***u2*** |  |  |  |  |  |  |  |  |  |
| TIAC1798 | 1 | 0 | 0 | 1 | 0 | 1 | 0 | 1 | 1 | 0 | 0 | 1 | 1 | 1 | 0 | 0 | 1 | 0 | 0 | 0 | 0 | 0 | 1 | 1 | 0 | 0 | 0 | 0 | 1 | **1** |
| TIAC1840 | 0 | 0 | 0 | 0 | 0 | 0 | 0 | 0 | 0 | 0 | 0 | 0 | 0 | 0 | 1 | 0 | 0 | 0 | 0 | 0 | 0 | 0 | 1 | 1 | 0 | 0 | 0 | 0 | 0 | 0 |
| TIAC1847 | 1 | 0 | 0 | 1 | 0 | 1 | 0 | 1 | 1 | 0 | 0 | 1 | 1 | 1 | 0 | 0 | 1 | 0 | 0 | 0 | 0 | 0 | 1 | 1 | 0 | 0 | 0 | 0 | 1 | **1** |
| TIAC1848 | 1 | 0 | 0 | 1 | 0 | 1 | 0 | 1 | 1 | 0 | 0 | 1 | 1 | 1 | 0 | 0 | 1 | 0 | 0 | 0 | 0 | 0 | 1 | 1 | 0 | 0 | 0 | 0 | 1 | **1** |
| TIAC1991 | 1 | 0 | 0 | 0 | 0 | 0 | 1 | 0 | 0 | 1 | 0 | 0 | 0 | 0 | 0 | 1 | 0 | 0 | 0 | 0 | 0 | 0 | 1 | 1** | 0 | 0 | 0 | 0 | 0 | 0 |
| TIAC1992 | 0 | 0 | 1* | 0 | 0 | 1* | 0 | 1 | 0 | 0 | 1 | 1 | 1 | 1 | 0 | 0 | 0 | 0 | 0 | 0 | 1 | 0 | 1 | 1** | 0 | 0 | 0 | 0 | 0 | 0 |
| TIAC1993 | 1 | 0 | 0 | 0 | 0 | 0 | 1 | 0 | 0 | 1 | 0 | 0 | 0 | 0 | 0 | 1 | 0 | 0 | 0 | 0 | 0 | 0 | 1 | 1** | 0 | 0 | 0 | 0 | 0 | 0 |
| TIAC1994 | 1 | 0 | 0 | 0 | 0 | 0 | 1 | 0 | 0 | 1 | 0 | 0 | 0 | 0 | 0 | 1 | 0 | 0 | 0 | 0 | 0 | 0 | 1 | 1** | 0 | 0 | 0 | 0 | 0 | 0 |
| TIAC2001 | 0 | 0 | 0 | 0 | 0 | 0 | 0 | 0 | 0 | 0 | 0 | 0 | 0 | 0 | 1 | 0 | 0 | 0 | 0 | 0 | 0 | 0 | 1 | 1 | 0 | 0 | 0 | 0 | 0 | 0 |
| TIAC3152 | 0 | 0 | 1 | 0 | 0 | 1 | 0 | 1 | 0 | 0 | 1 | 1 | 1 | 1 | 0 | 0 | 0 | 0 | 0 | 0 | 1 | 0 | 1 | 1** | 0 | 0 | 0 | 0 | 0 | 0 |
| TIAC3462 | 1 | 0 | 0 | 0 | 0 | 1 | 0 | 1 | 0 | 0 | 0 | 1** | 1 | 1** | 0 | 0 | 0 | 0 | 0 | 1 | 0 | 0 | 1 |  | 0 | 0 | 0 | 0 | 0 | 0 |
| TIAC3971 | 1 | 1 | 0 | 0 | 0 | 0 | 0 | 0 | 0 | 1 | 0 | 0 | 0 | 0 | 0 | 1 | 0 | 0 | 0 | 0 | 0 | 0 | 1 | 1 | 0 | 0 | 0 | 0 | 0 | 0 |
| TIAC3972 | 0 | 0 | 0 | 0 | 1 | 0 | 0 | 0 | 0 | 0 | 0 | 0 | 0 | 0 | 0 | 1** | 0 | 0 | 0 | 0 | 0 | 0 | 1 | 1** | 0 | 1 | 0 | 0 | 0 | 0 |
| SAMN02391177 | 0 | 0 | 0 | 0 | 0 | 1 | 0 | 1 | 0 | 0 | 0 | 1 | 1 | 1 | 0 | 0 | 0 | 0 | 0 | 0 | 1 | 0 | 1 | 1** | 1 | 0 | 1 | 1 | 0 | 0 |
| SAMN02403200 | 0 | 0 | 0 | 0 | 0 | 1 | 0 | 1 | 1 | 0 | 0 | 1 | 1 | 1 | 0 | 0 | 1 | 1 | 1 | 0 | 1 | 0 | 1 | 1** | 0 | 0 | 0 | 0 | 0 | 0 |
| SAMN13134218 | 0 | 0 | 0 | 0 | 0 | 1 | 0 | 1 | 0 | 0 | 0 | 1 | 1 | 1 | 0 | 0 | 0 | 0 | 0 | 1 | 0 | 0 | 1 | 1** | 0 | 0 | 0 | 0 | 0 | 0 |

The table shows the *se* gene profiles determined based on *in silico* PCR using all specific primer pairs described in literature (Supplementary table S1), and the amplicon alignment results to distinguish between *selu*, *selu2* and the pseudogenes *Ψent1* and *Ψent2*. Gene presence is indicated with ‘1’ in a green box, absence with ‘0’ in a pink box, as determined by all gene-specific primer pairs. *Not all gene-specific primer pairs (i.e. primer pairs with names as referred to in Supplementary Table S1: sec-3, sec-5 sec-10 and sec-12; and seg-2, seg-7, seg-8, seg-9 and seg-10) led to an *in silico* PCR product because of assembly fragmentation. **Not all gene-specific primer pairs (i.e. primer pairs with names as referred to in Supplementary Table S1: sem-1 and sem-3; seo-1 and seo-2; seq-6; selx-1 and selx-3) led to an *in silico* PCR product because of nucleotide variation within the primer binding sides, higher than accepted by the preset criteria (indicated in yellow in Supplementary Table S1).

Table S7. NCBI SRA accession numbers of WGS data of all isolates on BioProject ID PRJNA750393

| **Isolate Name** | **Accession number** |
| --- | --- |
| TIAC1840_GenElute-NL | SAMN20456185 |
| TIAC1848_GenElute-NL | SAMN20456186 |
| TIAC1991_GenElute-NL | SAMN20456187 |
| TIAC1992_GenElute-NL | SAMN20456188 |
| TIAC1993_GenElute-NL | SAMN20456189 |
| TIAC1994_GenElute-NL | SAMN20456190 |
| TIAC2001_GenElute-NL | SAMN20456191 |
| TIAC3462_GenElute-NL | SAMN20456192 |
| TIAC3971_GenElute-NL | SAMN20456193 |
| TIAC3972_GenElute-NL | SAMN20456194 |
| TIAC1798_GenElute-NL | SAMN20456195 |
| TIAC1798_GenElute | SAMN20456196 |
| TIAC1798_DNeasy | SAMN20456197 |
| TIAC1798_Wizard | SAMN20456198 |
| TIAC1847_GenElute-NL | SAMN20456199 |
| TIAC1847_GenElute | SAMN20456200 |
| TIAC1847_DNeasy | SAMN20456201 |
| TIAC1847_Wizard | SAMN20456202 |
| TIAC3152_GenElute-NL | SAMN20456203 |
| TIAC3152_GenElute | SAMN20456204 |
| TIAC3152_DNeasy | SAMN20456205 |
| TIAC3152_Wizard | SAMN20456206 |

The datasets supporting the conclusions of this study have been deposited in the NCBI SRA under the BioProject accession number PRJNA750393 (all in-house sequenced data). The accession number of each sample is shown. The sample names consist of the name of the respective isolate and an abbreviation of the applied DNA extraction kit, i.e. GenElute-NL: GenElute Bacterial gDNA kit using the protocol for Gram positive bacteria (without (i.e. no) lysostaphin, NL); GenElute: GenElute Bacterial gDNA kit using the protocol for Staphylococcal species (with lysostaphin); DNeasy: DNeasy Blood & Tissue kit; Wizard: Wizard gDNA Purification kit.

# References

Agilent Technologies (2015). Assay Quick Guide for 4200 TapeStation System. 3, 1–4.

Aguilar, J. L., Varshney, A. K., Wang, X., Stanford, L., Scharff, M., and Fries, B. C. (2014). Detection and measurement of staphylococcal enterotoxin-like K (SEl-K) secretion by *Staphylococcus aureus* clinical isolates. *J. Clin. Microbiol.* 52, 2536–2543. doi:10.1128/JCM.00387-14.

Aung, M. S., San, T., Aye, M. M., Mya, S., Maw, W. W., Zan, K. N., et al. (2017). Prevalence and genetic characteristics of *Staphylococcus aureus* and *Staphylococcus argenteus* isolates harboring panton-valentine leukocidin, enterotoxins, and TSST-1 genes from food handlers in Myanmar. *Toxins (Basel).* 9, 1–13. doi:10.3390/toxins9080241.

Aung, M. S., Urushibara, N., Kawaguchiya, M., Sumi, A., Takahashi, S., Ike, M., et al. (2019). Molecular epidemiological characterization of *Staphylococcus argenteus* clinical isolates in Japan: Identification of three clones (ST1223, ST2198, and ST2550) and a novel staphylocoagulase genotype XV. *Microorganisms* 7, 27–29. doi:10.3390/microorganisms7100389.

Bania, J., Dabrowska, A., Bystron, J., Korzekwa, K., Chrzanowska, J., and Molenda, J. (2006). Distribution of newly described enterotoxin-like genes in *Staphylococcus aureus* from food. *Int. J. Food Microbiol.* 108.

Bankevich, A., Nurk, S., Antipov, D., Gurevich, A. A., Dvorkin, M., Kulikov, A. S., et al. (2012). SPAdes: A New Genome Assembly Algorithm and Its Applications to Single-Cell Sequencing. *J. Comput. Biol.* 19, 455–477. doi:10.1089/cmb.2012.0021.

Becker, K., Roth, R., and Peters, G. (1998). Rapid and Specific Detection of Toxigenic Staphylococcus aureus : Use of Two Multiplex PCR Enzyme Immunoassays for Amplification and Hybridization of Staphylococcal Enterotoxin Genes, Exfoliative Toxin Genes, and Toxic Shock Syndrome Toxin 1 Gene. *J. Clin. Microbiol.* 36, 2548–2553. doi:10.1128/JCM.36.9.2548-2553.1998.

Bohach, G. A., and Schlievert, P. M. (1987). Nucleotide sequence of the staphylococcal enterotoxin C1 gene and relatedness to other pyrogenic toxins. *MGG Mol. Gen. Genet.* 209, 15–20. doi:10.1007/BF00329830.

Bolger, A. M., Lohse, M., and Usadel, B. (2014). Trimmomatic: a flexible trimmer for Illumina sequence data. *Bioinformatics* 30, 2114–2120. doi:10.1093/bioinformatics/btu170.

Chiang, Y. C., Liao, W. W., Fan, C. M., Pai, W. Y., Chiou, C. S., and Tsen, H. Y. (2008). PCR detection of Staphylococcal enterotoxins (SEs) N, O, P, Q, R, U, and survey of SE types in *Staphylococcus aureus* isolates from food-poisoning cases in Taiwan. *Int. J. Food Microbiol.* 121, 66–73. doi:10.1016/j.ijfoodmicro.2007.10.005.

Collery, M. M., and Smyth, C. J. (2007). Rapid differentiation of *Staphylococcus aureus* isolates harbouring *egc* loci with pseudogenes *ψent1* and *ψent2* and the *selu* or *selu v* gene using PCR-RFLP. *J. Med. Microbiol.* 56, 208–216. doi:10.1099/jmm.0.46948-0.

Couch, J. L., and Betley, M. J. (1989). Nucleotide sequence of the type C3 staphylococcal enterotoxin gene suggests that intergenic recombination causes antigenic variation. *J. Bacteriol.* 171, 4507–4510. doi:10.1128/jb.171.8.4507-4510.1989.

Couch, J. L., Soltis, M. T., and Betley, M. J. (1988). Cloning and nucleotide sequence of the type E staphylococcal enterotoxin gene. *J. Bacteriol.* 170, 2954–2970. doi:10.1128/jb.170.7.2954-2960.1988.

Cremonesi, P., Luzzana, M., Brasca, M., Morandi, S., Lodi, R., Vimercati, C., et al. (2005). Development of a multiplex PCR assay for the identification of *Staphylococcus aureus* enterotoxigenic strains isolated from milk and dairy products. *Mol. Cell. Probes* 19, 299–305. doi:10.1016/j.mcp.2005.03.002.

Etter, D., Schelin, J., Schuppler, M., and Johler, S. (2020). Staphylococcal Enterotoxin C—An Update on SEC Variants, Their Structure and Properties, and Their Role in Foodborne Intoxications. *Toxins (Basel).* 12, 584. doi:10.3390/toxins12090584.

Fitzgerald, J. R., Monday, S. R., Foster, T. J., Bohach, G. A., Hartigan, P. J., Meaney, W. J., et al. (2001). Characterization of a putative pathogenicity island from bovine *Staphylococcus aureus* encoding multiple superantigens. *J. Bacteriol.* 183, 63–70. doi:10.1128/JB.183.1.63-70.2001.

Fursova, K., Sorokin, A., Sokolov, S., Dzhelyadin, T., Shulcheva, I., Shchannikova, M., et al. (2020). Virulence Factors and Phylogeny of *Staphylococcus aureus* Associated With Bovine Mastitis in Russia Based on Genome Sequences. *Front. Vet. Sci.* 7, 1–10. doi:10.3389/fvets.2020.00135.

Heymans, F., Fischer, A., Stow, N. W., Girard, M., Vourexakis, Z., Courtis, A. Des, et al. (2010). Screening for Staphylococcal Superantigen Genes Shows No Correlation with the Presence or the Severity of Chronic Rhinosinusitis and Nasal Polyposis. *PLoS One* 5, e9525. doi:10.1371/journal.pone.0009525.

Holtfreter, S., Grumann, D., Schmudde, M., Nguyen, H. T. T., Eichler, P., Strommenger, B., et al. (2007). Clonal distribution of superantigen genes in clinical *Staphylococcus aureus* isolates. *J. Clin. Microbiol.* 45, 2669–2680. doi:10.1128/JCM.00204-07.

Jarraud, S., Cozon, G., Vandenesch, F., Bes, M., Etienne, J., and Lina, G. (1999). Involvement of enterotoxins G and I in staphylococcal toxic shock syndrome and staphylococcal scarlet fever. *J. Clin. Microbiol.* 37, 2446–2449. doi:10.1128/jcm.37.8.2446-2449.1999.

Jarraud, S., Mougel, C., Thioulouse, J., Lina, G., Meugnier, H., Forey, F., et al. (2002). Relationships between *Staphylococcus aureus* genetic background, virulence factors, agr groups (alleles), and human disease. *Infect. Immun.* 70, 631–641. doi:10.1128/IAI.70.2.631-641.2002.

Johler, S., Sihto, H.-M., Macori, G., and Stephan, R. (2016). Sequence Variability in Staphylococcal Enterotoxin Genes *seb*, *sec*, and *sed*. *Toxins (Basel).* 8, 169. doi:10.3390/toxins8060169.

Johnson, W. M., Tyler, S. D., Ewan, E. P., Ashton, F. E., Pollard, D. R., and Rozee, K. R. (1991). Detection of genes for enterotoxins, exfoliative toxins, and toxic shock syndrome toxin 1 in *Staphylococcus aureus* by the polymerase chain reaction. *J. Clin. Microbiol.* 29, 426–430. doi:10.1128/jcm.29.3.426-430.1991.

Letertre, C., Perelle, S., Dilasser, F., and Fach, P. (2003). Identification of a new putative enterotoxin SEU encoded by the *egc* cluster of *Staphylococcus aureus*. *J. Appl. Microbiol.* 95, 38–43. doi:10.1046/j.1365-2672.2003.01957.x.

Liang, C., Schaack, D., Srivastava, M., Gupta, S., Sarukhanyan, E., Giese, A., et al. (2016). A *Staphylococcus aureus* Proteome Overview: Shared and Specific Proteins and Protein Complexes from Representative Strains of All Three Clades. *Proteomes* 4, 8. doi:10.3390/proteomes4010008.

Mclauchlin, J., Narayanan, G. L., Mithani, V., and O’Neill, G. (2000). The detection of enterotoxins and toxic shock syndrome toxin genes in *Staphylococcus aureus* by polymerase chain reaction. *J. Food Prot.* 63, 479–488. doi:10.4315/0362-028X-63.4.479.

Mehrotra, M., Wang, G., and Johnson, W. M. (2000). Multiplex PCR for Detection of Genes for Staphylococcus aureus Enterotoxins, Exfoliative Toxins, Toxic Shock Syndrome Toxin 1, and Methicillin Resistance. *J. Clin. Microbiol.* 38, 1032–1035. doi:10.1128/JCM.38.3.1032-1035.2000.

Merda, D., Felten, A., Vingadassalon, N., Denayer, S., Titouche, Y., Decastelli, L., et al. (2020). NAuRA: Genomic Tool to Identify Staphylococcal Enterotoxins in *Staphylococcus aureus* Strains Responsible for FoodBorne Outbreaks. *Front. Microbiol.* 11, 1–12. doi:10.3389/fmicb.2020.01483.

Monday, S. R., and Bohach, G. A. (1999). Use of Multiplex PCR To Detect Classical and Newly Described Pyrogenic Toxin Genes in Staphylococcal Isolates. *J. Clin. Microbiol.* 37, 3411–3414. doi:10.1128/JCM.37.10.3411-3414.1999.

Nagaraj, S., Ramlal, S., Sripathy, M. H., and Batra, H. V. (2014). Development and evaluation of a novel combinatorial selective enrichment and multiplex PCR technique for molecular detection of major virulence-associated genes of enterotoxigenic *Staphylococcus aureus* in food samples. *J. Appl. Microbiol.* 116, 435–446. doi:10.1111/jam.12364.

Omoe, K., Hu, D. L., Takahashi-Omoe, H., Nakane, A., and Shinagawa, K. (2005). Comprehensive analysis of classical and newly described staphylococcal superantigenic toxin genes in *Staphylococcus aureus* isolates. *FEMS Microbiol. Lett.* 246, 191–198. doi:10.1016/j.femsle.2005.04.007.

Omoe, K., Ishikawa, M., Shimoda, Y., Hu, D. L., Ueda, S., and Shinagawa, K. (2002). Detection of *seg*, *seh*, and *sei* genes in *Staphylococcus aureus* isolates and determination of the enterotoxin productivities of *S. aureus* isolates harboring *seg*, *seh*, or *sei* genes. *J. Clin. Microbiol.* 40, 857–862. doi:10.1128/JCM.40.3.857-862.2002.

Ono, H. K., Omoe, K., Imanishi, K., Iwakabe, Y., Hu, D. L., Kato, H., et al. (2008). Identification and characterization of two novel staphylococcal enterotoxins, types S and T. *Infect. Immun.* 76, 4999–5005. doi:10.1128/IAI.00045-08.

Ono, H. K., Sato’o, Y., Narita, K., Naito, I., Hirose, S., Hisatsune, J., et al. (2015). Identification and characterization of a novel staphylococcal emetic toxin. *Appl. Environ. Microbiol.* 81, 7034–7040. doi:10.1128/AEM.01873-15.

Park, J. Y., Fox, L. K., Seo, K. S., McGuire, M. A., Park, Y. H., Rurangirwa, F. R., et al. (2011). Detection of classical and newly described staphylococcal superantigen genes in coagulase-negative staphylococci isolated from bovine intramammary infections. *Vet. Microbiol.* 147, 149–154. doi:10.1016/j.vetmic.2010.06.021.

Roetzer, A., Haller, G., Beyerly, J., Geier, C. B., Wolf, H. M., Gruener, C. S., et al. (2016). Genotypic and phenotypic analysis of clinical isolates of *Staphylococcus aureus* revealed production patterns and hemolytic potentials unlinked to gene profiles and source Clinical microbiology and vaccines. *BMC Microbiol.* 16, 1–14. doi:10.1186/s12866-016-0630-x.

Roussel, S., Felix, B., Vingadassalon, N., Grout, J., Hennekinne, J.-A., Guillier, L., et al. (2015). *Staphylococcus aureus* strains associated with food poisoning outbreaks in France: comparison of different molecular typing methods, including MLVA. *Front. Microbiol.* 6, 1–12. doi:10.3389/fmicb.2015.00882.

Schmitz, F.-J., Steiert, M., Hofmann, B., Verhoef, J., Hadding, U., Heinz, H.-P., et al. (1998). Development of a multiplex-PCR for direct detection of the genes for enterotoxin B and C, and toxic shock syndrome toxin-1 in *Staphylococcus aureus* isolates. *J. Med. Microbiol.* 47, 335–340. doi:10.1099/00222615-47-4-335.

Sharma, N. K., Rees, C. E. D., and Dodd, C. E. R. (2000). Development of a Single-Reaction Multiplex PCR Toxin Typing Assay for *Staphylococcus aureus* Strains. *Appl. Environ. Microbiol.* 66, 1347–1353. doi:10.1128/AEM.66.4.1347-1353.2000.

Shylaja, R., Murali, H. S., Batra, H. V., and Bawa, A. S. (2010). A novel multiplex PCR system for the detection of Staphylococcal enterotoxin B, *tsst*, *nuc* and *fem* genes of *Staphylococcus aureus* in food system. *J. Food Saf.* 30, 443–454. doi:10.1111/j.1745-4565.2010.00218.x.

Thomas, D. Y., Jarraud, S., Lemercier, B., Cozon, G., Echasserieau, K., Etienne, J., et al. (2006). Staphylococcal Enterotoxin-Like Toxins U2 and V, Two New Staphylococcal Superantigens Arising from Recombination within the Enterotoxin Gene Cluster. *Infect. Immun.* 74, 4724–4734. doi:10.1128/IAI.00132-06.

Tsen, H. Y., and Chen, T. R. (1992). Use of the polymerase chain reaction for specific detection of type A, D and E enterotoxigenic *Staphylococcus aureus* in foods. *Appl. Microbiol. Biotechnol.* 37, 685–690. doi:10.1007/BF00240750.

Vanneste, K., Garlant, L., Broeders, S., Van Gucht, S., and Roosens, N. H. (2018). Application of whole genome data for in silico evaluation of primers and probes routinely employed for the detection of viral species by RT-qPCR using dengue virus as a case study. *BMC Bioinformatics* 19, 312. doi:10.1186/s12859-018-2313-0.

Varshney, A. K., Mediavilla, J. R., Robiou, N., Guh, A., Wang, X., Gialanella, P., et al. (2009). Diverse enterotoxin gene profiles among clonal complexes of *Staphylococcus aureus* isolates from the Bronx, New York. *Appl. Environ. Microbiol.* 75, 6839–6849. doi:10.1128/AEM.00272-09.

Wilson, G. J., Seo, K. S., Cartwright, R. A., Connelley, T., Chuang-Smith, O. N., Merriman, J. A., et al. (2011). A novel core genome-encoded superantigen contributes to lethality of community-associated MRSA necrotizing pneumonia. *PLoS Pathog.* 7. doi:10.1371/journal.ppat.1002271.

1. <https://github.com/lh3/seqtk> [↑](#footnote-ref-1)
